# Architected Soft Actuators for Artificial Musculoskeletal Systems

**DOI:** 10.1002/adma.202501290

**Published:** 2025-07-24

**Authors:** Taekyoung Kim, Eliot A. Dunn, Melinda Chen, Ryan L. Truby

**Affiliations:** ^1^ Department of Materials Science and Engineering Northwestern University Evanston IL 60208 USA; ^2^ Center for Robotics and Biosystems Northwestern University Evanston IL 60208 USA; ^3^ Department of Mechanical Engineering Northwestern University Evanston IL 60208 USA

**Keywords:** 3D printing, architected materials, artificial muscles, soft actuators, soft robotics

## Abstract

Vertebrates depend on their musculoskeletal system for locomotion, manipulation, interaction with their environment, and more. The robustness and efficiency of animal locomotion are difficult to achieve in robots because their hardware does not replicate the mechanics and performance of animal bodies. Moreover, many state‐of‐the‐art soft actuators are ill‐suited as muscles in artificial musculoskeletal systems for deployable, task‐capable robots. This study presents an electrically‐driven, architected soft actuator that can be assembled into artificial musculoskeletal systems. The fully 3D printed actuators linearly extend and contract through the rotation of an integrated servo motor. They comprise a thermoplastic polyurethane handed shearing auxetic (HSA) and origami bellows structure. Together, these structures transmit torque, stretch, and resist torsional deflection in a manner that produces large linear actuation and force output up to 59 mm (or 30% strain) and 75 N, respectively. It showcases the actuator's performance as artificial muscles in a battery‐powered, human‐scale leg that can use three muscles to kick a ball. When accounting for the weight of auxiliary hardware, the actuators exhibit power and energy densities that are four orders of magnitude higher than for leading soft artificial muscles. The soft actuators represent a step toward providing robots with bioinspired musculoskeletal systems for animal‐like abilities.

## Graphical abstract

An architected soft actuator uses a servo motor and a combination of 3D printed architected elastomers to extend and contract. The actuator exhibits high actuation stroke, force output, and power density for constructing bioinspired artificial musculoskeletal systems. Human‐scale legs with bone‐inspired links, tendons, and three architected soft actuators as artificial muscles can kick and operate continuously on portable battery supplies.

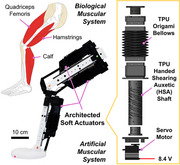

## 1 Introduction

Animals can easily adapt their bodies to navigate and perform within dynamic and unfamiliar environments. Robots cannot. While engineers construct robots from rigid, motorized mechanisms that allow for precise motion control, vertebrates leverage compliant, deformable, and imprecise musculoskeletal systems to produce complex motions and dynamically stable gaits that passively adapt to complex, unstructured conditions.^[^ ^1^, ^2^, ^3^ ^]^ The muscles, tendons, and bones of biological musculoskeletal systems work together to provide compliance, strength, and adaptability. Moreover, the elastic energy stored in muscle‐tendon units enables efficient movement and resilience to external perturbations.^[^ ^4^, ^5^, ^6^, ^7^ ^]^ Altogether, musculoskeletal systems enable a broad range of capabilities that are challenging to achieve in robots. These span the manual dexterity of humans, which supports both delicate and high‐force interactions during manipulation, to our climbing, swimming, and running abilities.^[^ ^8^, ^9^, ^10^ ^]^ Conventional robots may have actuators that can generate high forces, but they lack the mechanical compliance of muscle‐tendon units needed for mechanical adaptation^[^ ^11^, ^12^ ^]^ and the seamless integration between soft and rigid components. Thus, robots may struggle to replicate the same performance, robustness, and efficiency of movement observed in vertebrates without an equivalent musculoskeletal system.

In response to this need, recent efforts in soft and bioinspired robotics have aimed to develop robot bodies with muscle‐inspired soft actuators joined to rigid elements for force transmission.^[^ ^13^, ^14^, ^15^, ^16^, ^17^, ^18^, ^19^, ^20^ ^]^ Soft actuators for artificial musculoskeletal systems should provide four main capabilities. First, mechanically compliant and deformable soft actuators will enable adaptive movements. Soft actuators that provide high‐stroke and high‐force output will enable larger ranges of motion and work capacity. Soft actuators with a high specific power density will enable appropriate speed and performance for the overall artificial musculoskeletal system. Finally, soft actuators should facilitate seamless mechanical interfacing with rigid skeleton‐inspired features for force transmission. Ideal soft actuators for practical artificial musculoskeletal systems will also be inexpensive, easy to manufacture, electronically controllable, and scalable (i.e., to meet the force output and speed required for moving bone‐like elements).

Several classes of soft actuators popular in soft robotics provide the first two of these requirements.^[^ ^13^, ^14^, ^15^, ^16^, ^17^, ^18^, ^19^, ^20^ ^]^ Pneumatic artificial muscles (PAMs) can generate large actuation forces and high displacements as they expand and contract in response to changes in internal pressure. The muscle‐like movements, flexibility, and force outputs of PAMs have attracted attention for use in wearable robotic devices that assist human movement.^[^ ^21^, ^22^, ^23^ ^]^ Dielectric elastomer actuators (DEAs) quickly undergo large deformations in response to applied electric fields, enabling DEAs to replicate the fine, high‐bandwidth actuation capabilities of human muscle.^[^ ^24^, ^25^, ^26^, ^27^, ^28^, ^29^ ^]^ Shape memory alloy (SMA) actuators can undergo high‐force contractions in response to thermal stimuli. Their high specific work capacity has remained attractive for use in compact, lightweight robotic systems where space and weight are critical constraints.^[^ ^30^, ^31^, ^32^, ^33^ ^]^ Finally, motorized tendon‐driven mechanisms enable efficient, muscle‐like movements that are appropriate for applications where multiple degrees of freedom in actuation are necessary, such as in robotic arms and hands.^[^ ^34^, ^35^, ^36^, ^37^ ^]^ Depending on the motor and overall robot design, tendon‐driven actuators generate both large and fine movements.

Still, popular soft robotic actuators present limitations in their operating requirements, specific power density, and material properties for ease of integration with rigid bone‐like elements for force transmission^[^ ^38^, ^39^, ^40^, ^41^, ^42^, ^43^, ^44^, ^45^, ^46^ ^]^ (Table S1, Supporting Information). PAMs rely on heavy pumps and/or tanks to move working fluids like air in and out of the actuator to drive movement. They are frequently constructed from soft elastomers that are notoriously difficult to interface with rigid materials.^[^ ^38^, ^39^ ^]^ Although DEAs that operate at voltages below 1kV have been recently developed,^[^ ^40^ ^]^ most DEAs are still low‐force actuators that respond to electric potentials higher than 1 kV supplied by heavy, externally powered high‐voltage supplies. Driving motion with high voltages introduces safety concerns and requires specialized electronics that increase the overall complexity of a single actuator.^[^ ^41^, ^42^ ^]^ Recent SMA studies have tried to introduce active cooling methods to improve actuation speed.^[^ ^43^ ^]^ However, SMAs still require significant energy inputs for heating through thermal stimuli, resulting in low actuation bandwidth and efficiency, making them ill‐suited for applications requiring continuous or rapid movements.^[^ ^44^, ^45^ ^]^ In tendon‐driven actuation, controlling tendons requires precise tension control in multiple tendons simultaneously, which can be mechanically and computationally complex. Tendon materials also undergo wear and frictional loss over time that degrade performance and require careful maintenance.^[^ ^46^ ^]^

**Figure 1:**
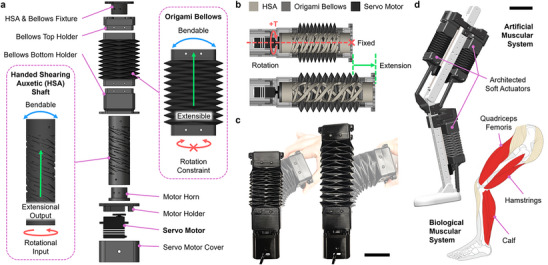
Design, Working Principle, and Application of Architected Soft Actuators. a) Architected soft actuators comprise an HSA shaft, origami bellows structures, and a servo motor. b) The diagram illustrates the working principle of actuation extension via servo motor rotation. c) Photographs show actuators at initial (left) and fully extended states (right); faded overlays show at‐rest and extended actuators bending under manual deformation. Scale bar is 50 mm. d) A robotic leg represents an artificial musculoskeletal system assembled from our architected soft actuators. The actuators serve as viscoelastic, artificial muscles that drive bone‐like linkages (top) in a manner reflecting a human leg (bottom). Scale bar is 100 mm.

Here, we report a motorized, architected soft actuator whose mechanical properties, operation, performance, and ease of fabrication result in an artificial muscle capable of powering human‐scale artificial musculoskeletal systems (**Figure** **1**). By integrating a common servo motor and two architected materials – a handed shearing auxetic (HSA) and a Yoshimura origami structure (Figure 1a) – we have achieved an electrically‐driven artificial muscle that linearly extends and contracts (Figure 1b). All components of the actuator, apart from the servo, are 3D printed from low‐cost thermoplastic elastomers that impart mechanical compliance, deformability, and robustness (Figure 1c). The entire mass of the actuator is 400 g and produces maximum actuation strokes of 29.5% ± 0.2% and maximum pushing and pulling forces of 74.5 ± 2.6 N and 75.2 ± 2.7 N, respectively. In a series of weightlifting and pulling exercises, the soft actuator demonstrated peak power densities of 12.3 W kg^−1^ while pushing a 5.65 kg load and 22.8 W kg^−1^ while pulling 6.78 kg – or 17 times the weight of the actuator. Our artificial muscle design enables seamless integration with elastomeric and rigid components, which we harness to construct a human‐scale robotic leg with three actuators and several rigid links that can be powered with a portable battery supply (Figure 1d). Overall, our architected soft actuators introduce movements, actuation capabilities, mechanical performance, and practical operating requirements that are not only difficult to achieve with existing soft actuators but streamline the construction of artificial musculoskeletal systems for bioinspired robots.

## 2 Results

### 2.1 Design and Working Principle of the Architected Soft Actuator

Our architected soft actuators are constructed from three primary components: a cylindrical HSA shaft, a Yoshimura origami bellows structure, and a servo motor (Figure 1a). Cylindrical HSAs have emerged as enabling materials for soft actuators directly driven by motors.^[^ ^46^, ^47^, ^48^, ^49^, ^50^, ^51^, ^52^ ^]^ They exhibit similar mechanical qualities to origami structures based on the Kresling pattern.^[^ ^53^ ^]^ Previous works have shown that when a torque is applied to one end of a cylindrical HSA shaft while its other end is constrained, the geometry undergoes a shearing motion such that the structure expands and extends in the longitudinal direction perpendicular to the applied torque.^[^ ^48^, ^49^ ^]^ In previous work, we used a rubber bellows shaft to transmit torque from a servo to one end of a single HSA to drive its extensional motion.^[^ ^52^ ^]^ However, the soft shaft buckled under large torques due to large torsional deflections, leading to a decrease in the force output from the actuator.

To create an architected soft actuator with higher torque transmission efficiency, we opted to use the HSA itself as a torque transmitting shaft and sought to create a constraining enclosure that was rotationally restrictive but still extendable under the extension of the HSA. We identified the Yoshimura origami structure as an appropriate architected material and designed it to be an outer bellows‐like structure. The Yoshimura pattern consists of alternating mountain and valley fold lines between triangular facets that maintain a balance between stiffness and flexibility.^[^ ^54^ ^]^ We shaped the Yoshimura pattern into a bellows structure that exhibits high longitudinal extensibility while resisting torsion. This allows the origami structure to constrain the rotation of the HSA shaft at its distal end while moving with the HSA as it actuates.

The HSA shaft and origami bellows are both 3D printed from elastomeric thermoplastic polyurethane (TPU) using fused deposition modeling (FDM). The fabrication process is illustrated in Figure S1 (Supporting Information) and described in detail in the Experimental Methods section. The actuator is robust to external shocks and durable due to the innate compliance of TPU. We designed the full actuator so that the HSA shaft ran through the center of the origami bellows. The HSA shaft is fixed to a servo motor at one end and to the origami bellows at the other. Thus, when the servo motor rotates, it applies a torque to the HSA shaft that transmits through the actuator to drive its linear motion (Figure 1b,c). The HSA shaft and origami bellows are still flexible and support actuation even under deformations like manual bending (Figure 1c). Multiple actuators can be printed and assembled as artificial muscles for a larger, integrated leg. For example, as shown in Figure 1d, the actuators can be mounted onto rigid bone‐like structures to move knee‐ and ankle‐inspired hinge joints.

### 2.2 Mechanical and Actuation Characterization

**Figure 2:**
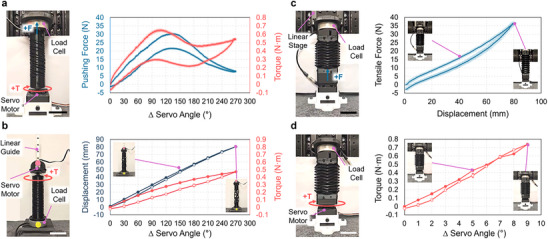
Mechanical Characterization of HSA Shaft and Origami Bellows. a‐d) The photographs at left show the experimental setups used to characterize the HSA shaft (a and b, left) and the origami bellows (c and d, left). The plots at right show corresponding characterization measurements: pushing force versus servo rotation angle (a, right) and displacement (b, right) for the HSA shaft, and tensile force versus displacement (c, right) and applied torque versus servo rotation angle (d, right) for the origami bellows. In all plots, closed and open circles represented loading and unloading response, respectively. Photographs in plots indicate specific points in the data. Error bars and shaded error bands indicate standard deviation (n  =  3). Scale bars are 50 mm.

Our architected soft actuator extends and contracts as the integrated servo motor rotates and transmits torques to the HSA shaft. The servo motors in our actuators can rotate from 0° to 270°: at 0°, the actuator is at rest, and at 270°, the servo is applying maximum torque to the actuator. Thus, an unconstrained actuator fully extends at 270° servo rotation. To understand how the HSA and Yoshimura bellows structure enable actuation, we characterized the mechanical, torque, and force responses of both components separately (**Figure** **2**).

We first characterized the blocked pushing force (Figure 2a) and free displacement (Figure 2b) of the HSA shaft during servo rotation to understand how it can extend the Yoshimura bellows. For both experiments, the HSA shaft was mounted to a 6‐axis load cell at one end to simultaneously measure the forces and torques generated; the other end was fixed to a servo motor. For blocked pushing force measurements, the HSA shaft was fixed at its initial length by a linear stage (Figure 2a‐left) and cycled through loading and unloading phases by repeatedly rotating the servo motor between 0° and 270° (Movie S1, Supporting Information). Rotating the motor produced a non‐monotonic pushing force and torque output, while unloading the actuator resulted in similar responses for both but with hysteresis (see Figure 2a‐right). The maximum pushing force was 30.3 ± 0.5 N at a 132° servo rotation angle. The HSA buckles at higher servo rotation, decreasing the force output to 7.7 ± 0.5 N at maximum servo rotation. A similar trend was observed in the HSA's torque response, which was a maximum of 0.65 ± 0.01 Nm at 109° servo rotation. For free displacement measurements, the servo was attached to a linear guide block to support its free motion during actuation (Figure 2b‐left; Movie S2, Supporting Information). We increased the servo angle from 0° to 270° and back at 30° increments. During loading, the HSA shaft linearly extended up to 80.4 ± 0.7 mm at maximum servo rotation of 270° (Figure 2b‐right). The torque generated during servo rotation also monotonically increased, and a torque of 0.47 ± 0.01 Nm was required at maximum rotation. The elastomeric nature of TPU facilitated full HSA shape recovery, and the nonlinear hysteretic responses observed in Figure 2b are expected given TPU's viscoelastic behaviors.

To understand how a twisting, torque‐transmitting HSA shaft can elongate an extensible but torque‐resistant element, we characterized the force‐extension (Figure 2c) and torque‐rotation response (Figure 2d) of the Yoshimura bellows. We performed a tensile test by cyclically stretching the bellows structure up to 80 mm at a 5 mm s^−1^ extension rate, which was the maximum free displacement we measured for the HSA shaft (Figure 2c‐left; Movie S3, Supporting Information). We used the same 6‐axis load cell to measure forces during bellows extension. As expected, the tensile force increased with extension, reaching a maximum force of 37.0 ± 0.3 N at full, 80‐mm extension (Figure 2c‐right). We also evaluated the bellows at higher strain rates of 10, 25, and 50 mm s^−1^ (see Figure S2, Supporting Information) and found that the force required to elongate the viscoelastic TPU structures increases with strain rate. Finally, we conducted a torque response test by loading and unloading the bellows structure as the servo rotated from 0° to 9° and back with 1° increments at a 1° s^−1^ rotation rate. The bellows structure was constrained at its initial length in this test (Figure 2d‐left; Movie S4, Supporting Information). The results in Figure 2d‐right show that applied torque monotonically increases with increasing servo angle, reaching a maximum torque of 0.74 ± 0.01 Nm at 9°. Thus, since the HSA shaft generates ≈0.5 Nm of torque at maximum servo rotation (see Figure 2b‐right), we expect that the Yoshimura bellows will experience less than 9° of torsional deflection. We confirm in Figure S3, Supporting Information, that the Yoshimura bellows is radially stiff but axially compliant (i.e., we show that the force required to rotate the bellows over a 6‐mm arc length is approximately six times higher than that required to elongate the same distance). Once again, since the Yoshimura bellows are also 3D printed from TPU, we observe the expected hysteretic responses in the data in Figure 2c. The torque response of the TPU Yoshimura bellows was also hysteretic but to a lesser extent compared to the tensile extension response.

**Figure 3:**
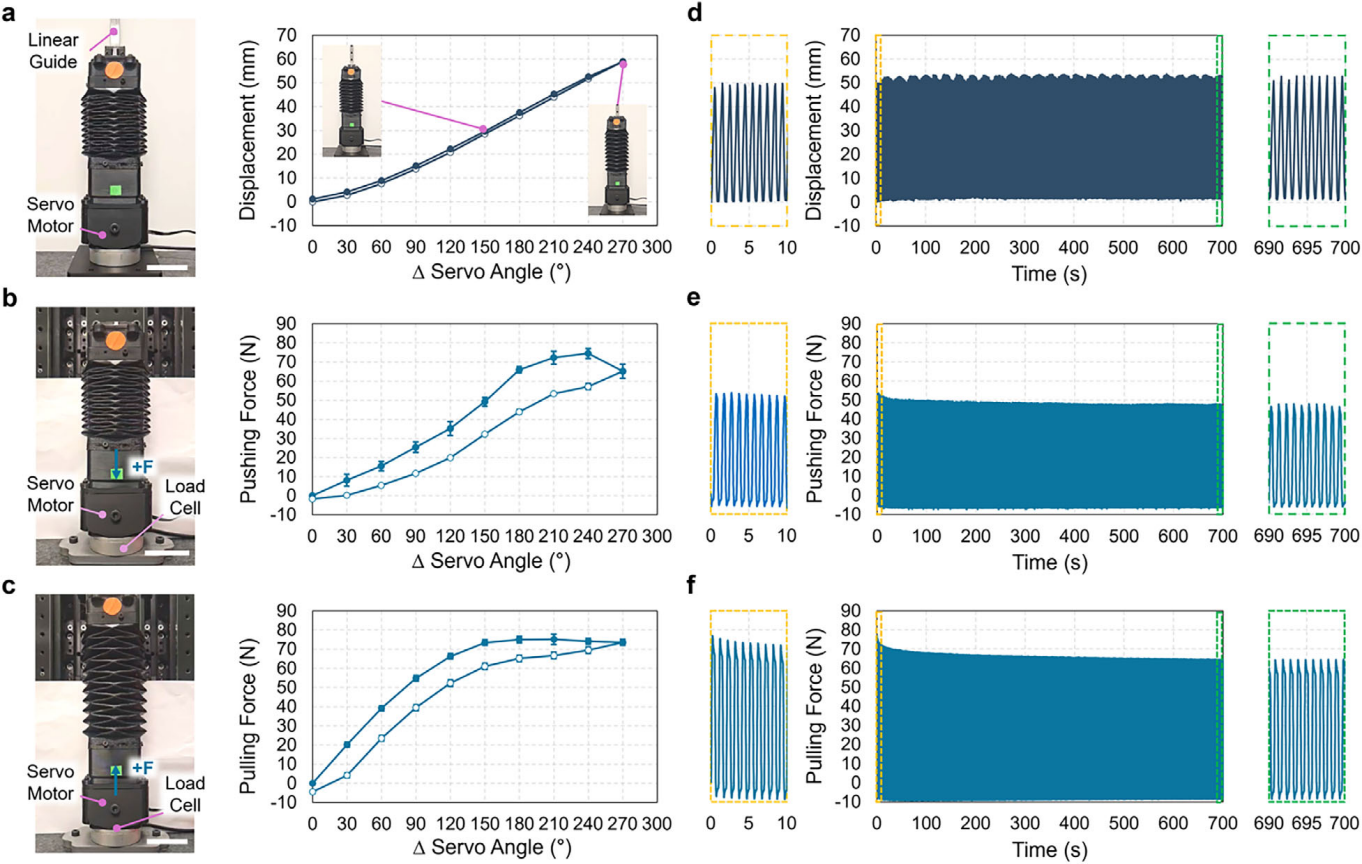
Architected Soft Actuator Displacement and Force Output. a‐c) The photographs show the experimental setups used to measure the extensional displacement (a, left), blocked pushing force (b, left), and blocked pulling force of the actuators (c, left). The accompanying plots provide displacement (a, right), pushing force (b, right), and pulling force (c, right) versus servo angle. In all plots, closed and open circles indicate loading and unloading data, respectively. The plots in (d) to (f) show reliability test results for each actuation case in (a) to (c); displacement (d), pushing force (e), and pulling force (f) are provided over 700 actuation cycles. The yellow and green insets show results at the beginning and end of the reliability tests, respectively. Error bars and shaded error bands indicate standard deviation (n  =  3). Scale bars are 50 mm.

As shown in **Figure** **3**, we characterized the displacement and force output range of the fully assembled architected soft actuator. We performed free displacement experiments using the same methods as for the freely actuating HSA shaft (Figure 3a‐left; Movie S5, Supporting Information). The actuator linearly extended 58.9 ± 0.4 mm at a maximum servo rotation of 270° (Figure 3a‐right). This corresponds to a 29.5% actuation strain with respect to the actuator's initial length of 200 mm. The actuator's maximum stroke is ≈25% less than that which we measured for the rail‐mounted HSA shaft (Figure 2b) because the bellows structure restricts HSA extension.

We then evaluated the pushing force generated by the actuator (Movie S6, Supporting Information). For these tests, a restraint was installed to the top of the actuator and connected to a linear stage to restrict extension during servo rotation (Figure 3b‐left). The blocked pushing force was measured using a load cell at the bottom end of the actuator. Figure 3b‐right presents the pushing force as a function of the servo rotation angle, showing a nonlinear, hysteretic response due to the viscoelastic properties of the HSA shaft and origami bellows. The pushing force gradually increased until a maximum of 74.5 ± 2.6 N was reached at 240° servo rotation. We attribute the decreasing force output for servo angles above 240° to the HSA shaft buckling inside the bellows, which reduces its capacity to generate force.

We also evaluated the blocked pulling force generated by the actuator (Movie S7, Supporting Information). For these tests, the actuator was fully extended by rotating the servo to 270° and then constrained (Figure 3c‐left); the blocked pulling force was measured with a load cell fixed to the bottom end of the actuator as the servo was rotated from 270° to 0° and back. We observed that the pulling force gradually increased as the servo rotated back to 0°, reaching a maximum pulling force of 75.2 ± 2.7 N at 60° (Figure 3c‐right). Again, the force response is nonlinear and hysteretic.

Altogether, our results show that the assembled actuator generates a higher maximum blocked pushing force than that of the HSA shaft alone: 74.5 ± 2.6 N (Figure 3b) versus 30.3 ± 0.5 N (Figure 2a). We expect this difference arises due to torsional buckling in the HSA at large servo rotation angles (recall Movie S1, Supporting Information). It also implies that the actuator's origami bellows can prevent severe buckling of the HSA shaft. The actuator also generated a higher maximum blocked pulling force than that of the HSA alone: 75.2 ± 2.7 N (Figure 3c) versus 48.8 ± 0.7 N (Figure S4 and Movie S8, Supporting Information). We attribute this difference to the stored elastic energy of the extended origami bellows. We also observed during the actuator characterization experiments that the axial stiffness of the actuator increases when actuated. Figure S5 (Supporting Information), shows through compression testing of the actuator that it becomes stiffer at increasing actuation strains. This dynamic stiffening effect is analogous to the dynamic stiffening of muscle during activation and may prove advantageous for more effectively transmitting force and maintaining system stability during operation.

Lastly, we conducted durability tests over 700 cycles of actuation in the free displacement (Figure 3d), pushing force (Figure 3e), and pulling force (Figure 3f) setups. Free displacement tests were conducted by rotating the servo motor between 0° and 270° at ≈1 Hz to cyclically extend the actuator as quickly as possible (Movie S9, Supporting Information). For the pushing and pulling force tests, the servo motor was rotated between 0° and 150° at ≈1 Hz. Collectively, Figure 3d‐f show that actuation performance remains stable over >700 cycles of actuation. Figure S7 (Supporting Information), also shows stable actuation performance over 700 cycles at 2 and 5 Hz actuation frequencies. As expected, the overall peak displacement and force output for the actuator decrease with increasing actuation frequency.

### 2.3 Architected Soft Actuators as Artificial Muscles

**Figure 4:**
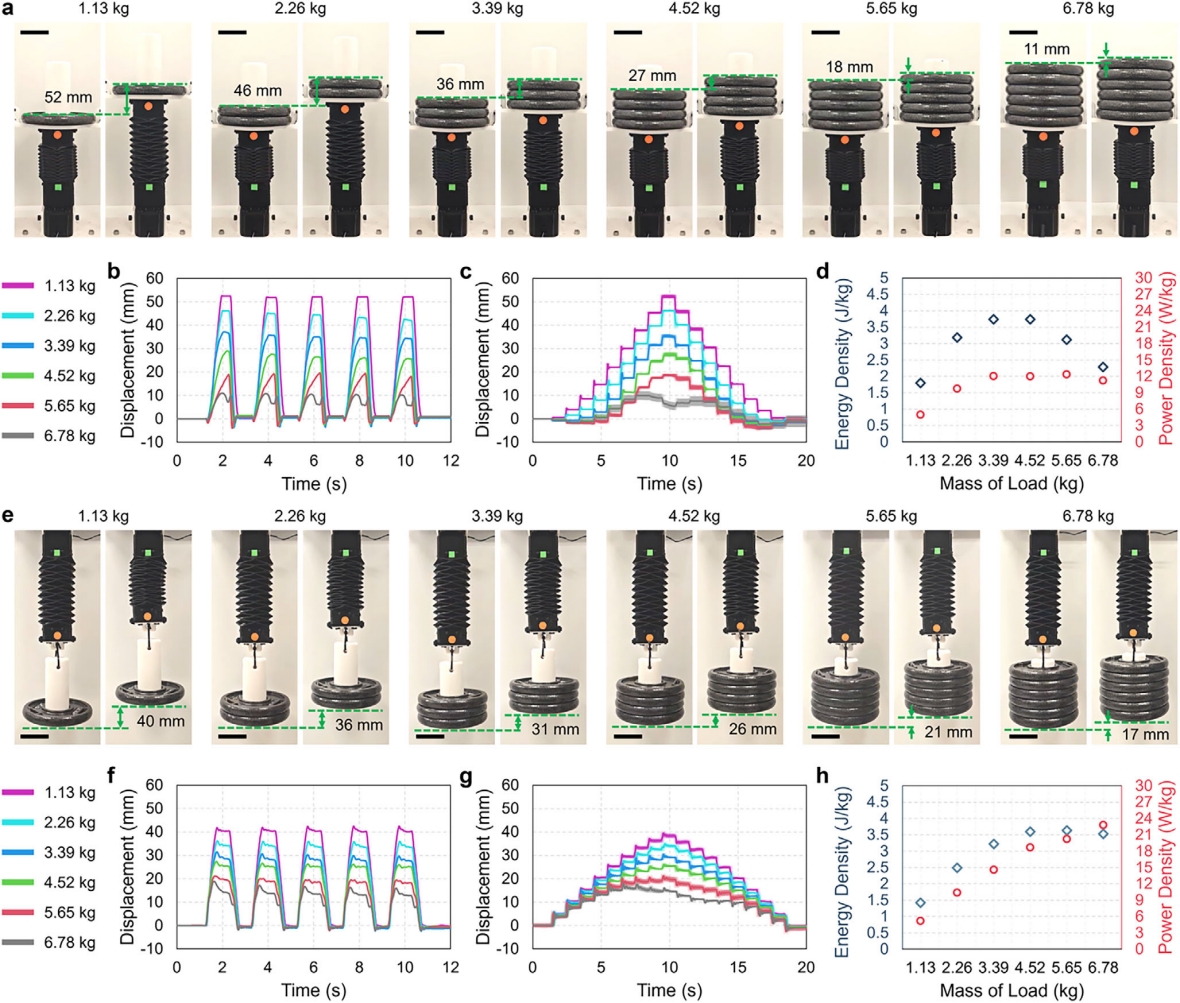
Pushing and Pulling Under Loads. a‐d) and e‐h) correspond to characterization data for actuation pushing and pulling, respectively. (a) Photographs of an architected soft actuator pushing six different loads; each pair shows an actuator in initial (left) and extended states (right). (b) Pushing displacement data during five lifts of each mass (from servo rotations between 0° and 270°) are plotted versus time. (c) Pushing displacement data during 30° increment servo rotations from 0° to 270° are provided over time for lifting each mass. (d) Energy and power densities are provided for each lifted load. (e) Photographs of an architected soft actuator pulling the same six loads from (a); each pair shows an actuator in extended (left) and initial states (right). (f) Pulling displacement data during five pulls of each mass are plotted versus time. (g) Pushing displacement data are provided over time for pulling each mass. (h) Energy and power densities are provided for each pulled load. Shaded error bands indicate standard deviation (n  =  3). Scale bars are 50 mm.

To demonstrate the use of our architected soft actuators as muscles for artificial musculoskeletal systems, we analyzed their performance while pushing and pulling various weights (Movie S10, Supporting Information). In these experiments, two linear guides directed the actuators in pushing trials to ensure their balance during weightlifting, and a 3D printed fixture was installed on top of the actuator to load up to six, 1.13 kg weights. The actuator lifts weights as the servo motor rotates to 270° at maximum speed; it resets as the servo rotates back to 0°. **Figure** **4**a shows the actuators at their maximum pushing stroke when 1 to 6 weights (i.e., 1.13–6.78 kg) are loaded. As shown in Figure 4b, the maximum stroke output decreased with increasing load, and the time required to reach the maximum stroke increased. The actuator also returned to its rest state more quickly as the servo was rotated back to 0° because larger loads compressed the actuator and counterrotated the servo. We also observed that the actuator can only lift the maximum weight of 6.78 kg to ≈10 mm (i.e., 5% strain). This load compressed the actuator such that it could not fully extend, even as the servo provided increasing torque to the HSA shaft. Figure 4c shows the actuator's displacement under the six different loads as the servo rotated through its full range at 30° increments held for 1 s. For the 1.13 to 5.65 kg trials, the maximum displacement for each servo rotation decreased nearly linearly with increasing load. The 6.78 kg load was not lifted as expected, with increased servo rotation not producing an increase in stroke. Lastly, we calculated the actuator's peak energy and power densities under each load based on the maximum stroke and the time required to reach it. Peak energy density increased with load up to 3.7 J kg^−1^ at 4.52 kg, after which it decreased with increasing load. Peak power density followed a similar trend, reaching 12.3 W kg^−1^ at 5.65 kg, and then plateaued for increasing weight.

The actuator can pull weights when hung upside down and actuated from extension at 270° servo rotation to 0°. We evaluated the actuator's performance while pulling the same range of weights by rotating the servo at maximum speed. Figure 4e shows the actuator's maximum pulling strokes under each load. As the load increased, the maximum pulling stroke decreased nearly linearly (Figure 4f). The load of 6.78 kg again represents a maximum that can be pulled by the actuator and still produces an expected displacement profile in response to step‐wise servo inputs. Like Figure 4c,g shows the actuator's displacement under the six different loads as the servo rotated through its full range at 30° increments held for 1 s. Again, the actuator did not lift the 6.78 kg load in the expected profile since the weight stretched the actuator; the servo could not provide sufficient torque to counteract the added load. Lastly, Figure 4h shows the actuator's energy and power densities during pulling. Peak energy density increased up to 3.6 J kg^−1^ at 5.65 kg and only slightly decreased with increasing load. By contrast, the power density monotonically increased for all loads, reaching 22.8 W kg^−1^ at 6.78 kg, about twice that measured for pushing. We attribute the noticeable differences in the actuator's energy and power densities during pushing and pulling to how applied loads deform the actuator. In the pulling case, applied loads increase the elastic energy stored in the actuator, which facilitates quick returns to the actuator's extended state as the servo rotates from 0° to 270°.

We used the data in Figure 4b,d to calculate the minimum energy efficiency of our actuators based on (i) the voltage and current values at which the servo motor can provide its stall torque, (ii) the time it takes the actuator to provide the maximum stroke, and (iii) the actual pushing or pulling displacements under different load conditions (see Figure S8, Supporting Information). The energy efficiencies of pushing and pulling increase up to 6.4 ± 0.2% for the 3.39 kg load and 7.6 ± 0.1% for the 4.52 kg load, respectively. Above these loads, energy efficiency during pushing decreases while efficiency during pulling remains steady.

Finally, to test the actuator's robustness, we conducted a durability test as the actuator repeatedly pulled a weight of 1.13 kg for 10 000 cycles at 1 Hz (Movie S11, Supporting Information). We compared the actuator's pulling stroke at the 1st, 5000th, and 10 000th cycles and observed a 10% change in the maximum actuation stroke at the 10 000th cycle. This is likely due to the TPU stretching over time due to creep. In the future, several strategies can be considered during the design and manufacturing stages to address this creep issue. For instance, adjusting the actuator geometry to reduce localized stress concentrations or incorporating reinforcement structures can help mitigate creep effects. Overall, the actuator operated without malfunction over 10 000 cycles, demonstrating its robustness. We did not observe any failures in the HSA shaft, origami bellows, or the interface with the rigid servo motor. A slight wobble was sometimes observed at the connection between the servo motor and the mounting plate that can be reduced in future designs with adhesives and other permanent fastening methods.

### 2.4 Artificial Musculoskeletal Systems

**Figure 5:**
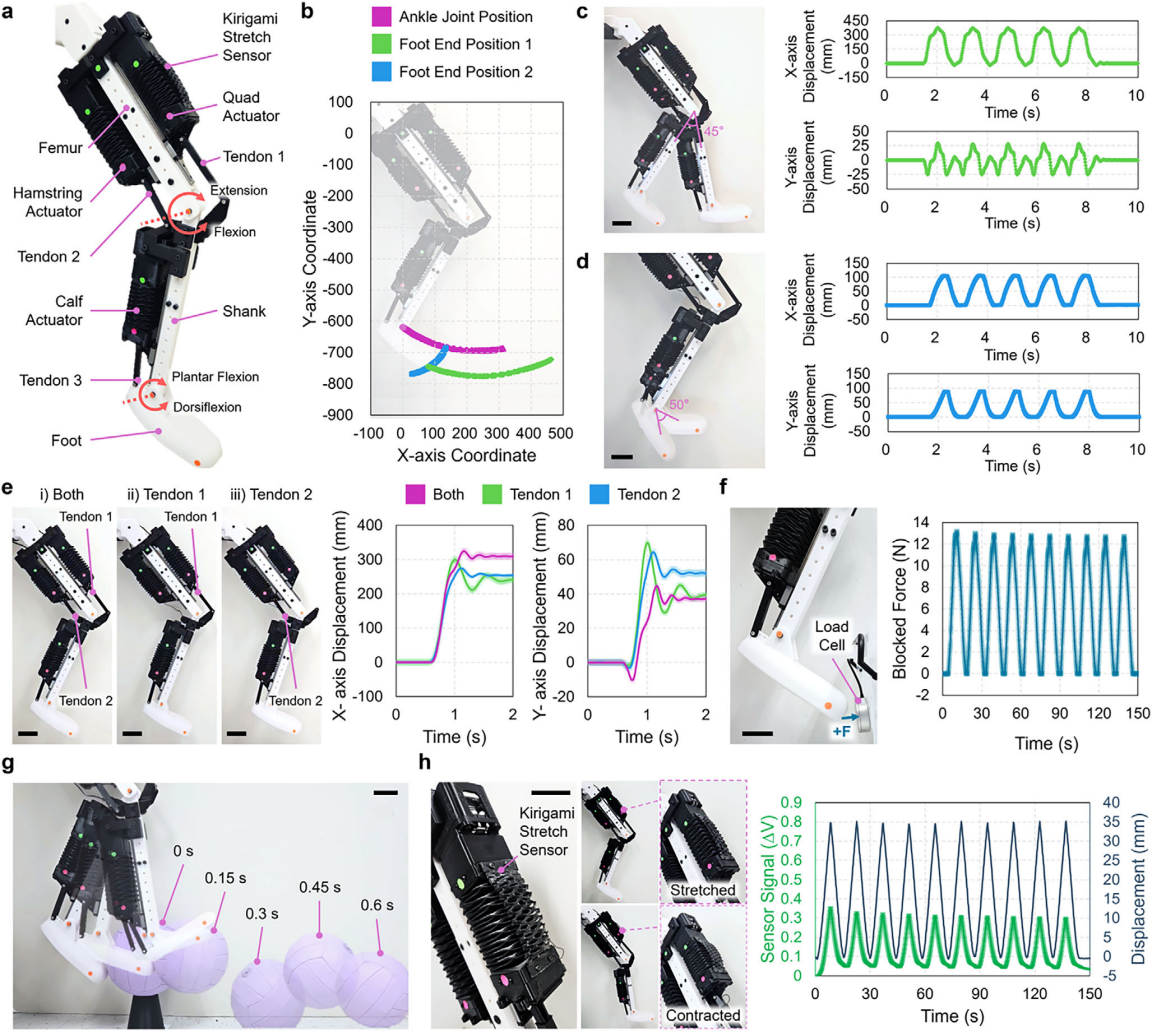
A Robotic Leg as an Artificial Musculoskeletal System. a) An artificial robotic leg consists of three artificial muscles (i.e., quad, hamstring, and calf actuators), three tendons, and PLA skeletal and foot structures. b) The ranges of motion achieved by moving the three actuators are indicated with respect to the initial leg position shown in the faded photograph. The ankle joint and Foot End Position 1 trajectories are generated by knee extension and flexion; the Foot End Position 2 trajectory is generated by ankle dorsiflexion and plantar flexion. c,d) The overlaid photographs indicate the full range of knee (c, left) and ankle (d, left) motions. The accompanying plots provide knee (c, right) and ankle (d, right) joint displacements in the x, y frame over time during five cycles, the full range of each joint motion. e) Photographs of system response test cases during knee extensions (left) with various tendon connections: i) the quad and hamstring actuators are both connected to their tendons (Tendons 1 and 2); ii) only Tendon 1 is connected; and iii) only Tendon 2 is connected. A plot of the corresponding displacement responses in the x‐ and y‐ axes is provided. f) The photograph illustrates the blocked force test set‐up using a load cell (left), and a plot of blocked force (F) by the full range of knee motion is provided for ten actuation cycles (right). g) A time‐lapse photograph shows the leg's motion as it kicks a ball; overlaid photographs are shown at 0.15‐s intervals. h) The Kirigami stretch sensor is mounted on the quad actuator; the photographs at left show the sensor integrated with the quad actuator and in stretched and contracted states. The proprioceptive feedback provided by the sensor is shown in the plot of the sensor voltage signal and the quad actuator's displacement versus time (right). Shaded error bands indicate standard deviation (n  =  3). Scale bars are 50 mm.

The overall design and performance of our architected soft actuators streamlined their integration as muscles into artificial musculoskeletal systems. We constructed a human‐scale robotic leg complete with knee and ankle‐like hinge joints, kicking capabilities, and proprioception from a soft sensor (**Figure** **5**). The leg comprises three architected soft actuators for artificial muscles, skeletal features for bone‐inspired links, and tendon‐inspired connectors (Figure 5a). We 3D printed the rigid bone links from PLA filament and the tendon‐inspired connectors from TPU. We refer to the three artificial muscles as the quad, hamstring, and calf actuators and the two bone features as the femur and shank links. The quad and hamstring muscles actuate along linear guides fixed to both the anterior and posterior sides of the femur link, and we installed the calf actuator to a linear guide on the posterior side of the shank. The linear guide rails prevent the actuators from bending, maintain the directionality of contraction or extension, and ensure effective force transmission to rotate the joints. We used TPU tendons to connect the quad and hamstring muscles to the shank link and the calf muscle to the foot structure. The robotic leg replicates knee extension and flexion through the cooperative pushing and pulling from the quad and hamstring actuators, as well as dorsiflexion and plantar flexion of the ankle through pushing and pulling by the calf actuator. The TPU tendons transmit forces from the architected soft actuators, enabling hinge joint motions. The total leg weighs 2.9 kg and its length is 890 mm.

We evaluated the leg's range of motion via the actuation of its three artificial muscles. Movie S12 (Supporting Information) shows that alternating between full extension and contraction of the quad and hamstring actuators drives alternating knee extension and flexion. Repeatedly contracting and extending the calf actuator from its initial state similarly rotated the foot about the ankle. We placed colored markers at the proximal end of the femur link, the ankle joint, and the foot end to extract ankle joint and foot end trajectories from video data (Figure 5b). From these trajectories, the knee sweeps 45° between maximum flexion and extension, and the ankle moves 50° between maximum dorsiflexion and plantar flexion. Figures 5c,d show the displacement of the foot end in x‐y coordinates for five extension‐flexion cycles of the knee and five dorsiflexion‐plantar flexion cycles of the ankle. Overall, the actions of the artificial muscles drive consistent motion in the lower leg and foot. Figure S9 (Supporting Information), shows that the actuators are still capable of moving the leg even when disconnected from the linear guides. The actuators stretch and bend slightly without the rails, and the foot hangs ≈1 cm lower as a result. However, the range of leg motion during knee bending is unchanged. We also show that knee bending angle can be estimated by a fourth‐order polynomial of servo rotation angle. The derivation process and results are presented in Note S1, Supporting Information.

Tendons and ligaments serve critical mechanical functions in biological musculoskeletal systems, including joint stabilization and the storage and release of elastic energy for efficient motion (7). The collective viscoelastic mechanical properties of muscles, tendons, and ligaments also enable mechanical adaptation and dampening of external forces acting on an entire musculoskeletal system (5). These qualities – which are challenging, if not impossible, to replicate in traditional robots that move using electromagnetic motors – are essential to the efficiency and robustness of vertebrate locomotion (3), (4), (8). To understand how our architected soft actuators impart bioinspired dampening and force transmission capabilities to artificial musculoskeletal systems, we performed a range of movements with the knee to test the leg's mechanical response (Movie S13, Supporting Information). We first analyzed how the tendons influence the lower leg's motion during step extensions (Figure 5e). When both tendons are connected, and the artificial muscles actuate at maximum speed, the knee extends, and the lower leg stabilizes with a minimum overshoot in displacement. Removing either Tendon 1 or Tendon 2 (i.e., the tendons connected to the quad and hamstring muscles, respectively) results in a smaller displacement and a more underdamped response to the knee extension, as shown in Figure 5e. A step‐actuation like those shown produces a brief period of dynamic, oscillatory bending because the architected soft actuators are viscoelastic elements themselves. A single soft actuator can stabilize this dynamic motion within seconds, but removing a tendon prevents the other muscle from dampening this response even more quickly. We also measured the blocked pushing force at the foot end during cyclic knee extensions (Figure 5f‐left, Movie S14, Supporting Information). We measured consistent force outputs up to a maximum of 13.1 ± 0.1 N over ten extension‐bending cycles (Figure 5f‐right). The leg's force output at the foot is lower than the blocked forces produced by an individual quad or hamstring actuator. This results from the muscles acting on a longer moment arm (i.e., they must move the entire lower leg) and the mechanical compliance that the muscles and tendons introduce to the entire leg assembly.

We used our insights into the leg's performance and mechanical behaviors to kick a ball with a motion generated by all three artificial muscles (Movie S15, Supporting Information). We started the leg in a position of maximal knee flexion and ankle plantar flexion. The artificial muscles moved at maximum servo operation speed to extend the knee and dorsiflex the ankle simultaneously. This generated sufficient force to kick a volleyball off a stand (Figure 5g).

All functional demonstrations with the robotic leg were achieved through open‐loop control. However, like their biological equivalents, artificial musculoskeletal systems will require proprioceptive sensing capabilities to provide kinematic feedback.^[^ ^55^ ^]^ We equipped the leg with a stretchable Kirigami strain sensor to demonstrate that our architected soft actuators facilitate easy integration of proprioceptive sensing capabilities (Figure 5g). Kirigami structures, another class of architected materials, can accommodate large strains through their cut pattern.^[^ ^56^ ^]^ We constructed a sandwich‐structured Kirigami soft sensor by laminating a 3D‐printed conductive TPU layer between two 3D printed non‐conductive TPU layers. The Kirigami sensor moves along with the motion of the architected soft actuator, reversibly stretching to accommodate muscle contractions and extensions (Figure 5g‐left). The conductive TPU layer is piezoresistive, and its resistivity increases under strain.^[^ ^57^ ^]^ We used a voltage divider and analog‐digital converter (ADS1115, Adafruit) to record voltage response from the Kirigami sensor during ten cycles of repeated 35‐mm extensions of the quad muscle. We observed a tight coupling of the sensor voltage output and quad extension (Figure 5g‐right; Movie S16, Supporting Information).

Finally, we conducted a battery‐powered motion test to demonstrate that our artificial musculoskeletal system is durable and can be practically operated with a portable power source (Movie S17, Supporting Information). We powered the leg with an 8,200 mAh LiPo battery (8.37 V, 99% charged) and completed knee extension‐bending cycles at 1 s for 1 h, operating the quad and hamstring actuators to their full servo rotation range and speed. The lower leg moved 3,600 cycles in 1 h and showed no signs of mechanical failure; the battery's final voltage and capacity values were 7.92 V and 70%, respectively. It confirms that the leg can be operated in this way for ≈3 h with a single battery, though the leg's performance decreases as the battery capacity decreases over time. This demonstrates the practicality of our artificial musculoskeletal system, overcoming the portability challenges faced by other soft actuators that respond to environmental stimuli or require power sources, such as high‐voltage equipment, pumps, or compressed gas tanks, that can be prohibitively heavy or impossible to disconnect from wall power.

## 3 Discussion

We have developed an electrically‐driven soft actuator with a design, ease of fabrication, and capabilities necessary to perform as artificial muscles in artificial musculoskeletal systems. Our actuators comprise two architected soft materials, a handed shearing auxetic and a Yoshimura bellows structure, that exhibit durability, extensibility, and anisotropic stiffness. Together, the elastomeric HSA and Yoshimura structures produce linear motions through the turn of a servo motor. Both structures are fabricated from thermoplastic elastomers using desktop FDM 3D printing, which is widely accessible to most engineers and hobbyists.^[^ ^58^ ^]^ While both the HSA and Yoshimura structures have been used in prior works for different applications, their synergistic use in this work gave rise to motorized soft actuators that neither structure alone could achieve. For example, our actuators can both pull and – unlike biological muscle – push loads up to 17 times greater than their nominal weight. Our actuator's design allowed us to bypass unwieldy auxiliary hardware required for other soft actuators, which pose systems‐level challenges toward developing deployable, untethered robots.^[^ ^59^, ^60^ ^]^ We achieved human leg‐like motions in an artificial musculoskeletal system comprising multiple artificial muscles, bone‐like rigid links, and elastomeric tendons. Our leg exhibited knee extension and flexion, ankle dorsiflexion and plantar flexion, and kicking abilities using the range of motion and force output the artificial muscles provided. Finally, as we look toward controlling the motion of artificial musculoskeletal systems, our actuators’ design facilitated the incorporation of a soft sensor that can support feedback control.

Compared to other leading artificial muscles, our soft actuators present several advantages, which we outline in Table S1 (Supporting Information). They provide contractile and extension motions in one system, which is difficult, if not impossible, to achieve in other artificial muscles. Electrostatic actuators require large voltages to operate (e.g., 1–20 kV), and pneumatic actuators can use pumps and compressors that require wall power (e.g., 110 V AC). Our motorized soft actuators use a low voltage (<10 V) input. They generate larger maximum output forces than electrostatic actuators but lower output forces than pneumatic ones. To achieve these capabilities, though, our artificial muscles require an integrated servo motor, the complex forms of the HSA and Yoshimura structure, and the design we have described here. We also compare our soft actuators’ performance to biological and artificial muscles in Table S2 (Supporting Information). First, our actuator shows a maximum linear displacement of 30% in contraction and extension, which is comparable to that of mammalian skeletal muscle (40%). The maximum power and energy densities of muscle (200 W kg^−1^ and 40 J kg^−1^) are still an order of magnitude higher than our actuators’ (40 W kg^−1^ and ≈4 J kg^−1^).

In Table S2 (Supporting Information), we provide the reported peak power and energy densities for selected artificial muscle technologies. However, reported values in the literature are determined based on the material mass of the soft actuator itself; the weights of the essential power sources are rarely accounted for in these values, if reported at all. Future untethered robots constructed from artificial musculoskeletal systems will need to incorporate power sources and auxiliary components on board. Thus, a more meaningful measurement of artificial muscle power and energy densities would therefore account for the additional hardware needed for actuation. In Table S3 (Supporting Information), we provide the peak power and energy densities of the same artificial muscle technologies but accounting for the actuator's nominal weights (i.e., the sum of the material weight and reported power source weight). When nominal weights are considered, we find that our soft actuators exhibit power and energy densities that are four orders of magnitude higher than those of other soft actuators. This points to our actuators’ potential as a practical, simple‐to‐implement approach toward constructing artificial musculoskeletal systems for untethered, more energy‐efficient, and robust robots. This comparison is also summarized in Figure S11 (Supporting Information).^[^ ^22^, ^24^, ^27^, ^29^, ^61^ ^]^

In closing, our work opens new avenues to the design and fabrication of vertebrate‐inspired musculoskeletal systems for deployable, robustly locomoting robots. Our work also creates several opportunities for further investigation, including design optimization of the architected soft actuators to improve their power density and learning how bioinspired robots can control limbs complete with muscles, tendons, and bones. We anticipate that the soft actuation strategy we have introduced can help engineers close the performance gap between robots and animals by providing robots with smoother motions, more adaptive gaits, and more economical locomotion required for operating in unstructured real‐world environments.

## 4 Experimental Section

### Actuator Design and Fabrication

HSA shafts and origami bellows were designed in Onshape (PTC) and Solidworks 2023 (Dassault), respectively. Thin, reinforcing wall structures were added to the HSA shaft between the gaps of its auxetic pattern to increase stiffness and force output. The reinforcing in‐fill improved HSA performance (Figure 2a) compared to HSAs printed without them (Figure S12, Supporting Information); more details are provided in Note S2 of the Supporting Information. 3D models of HSAs and origami bellows were converted to 3D printing sketch files using a commercial slicer (Bambu Studio, Bambu Labs). All parts in this work were 3D printed using an FDM 3D printer (X1 Carbon, Bambu Labs). HSAs were 3D printed from thermoplastic polyurethane (TPU, TPU 95 A Black, SUNLU) with a length of 130 mm (including a 90 mm long auxetic pattern region), a thickness of 4.5 mm, and inner and outer diameters of 31 and 40 mm, respectively. Origami bellows were also 3D printed from TPU (TPU 95 A Black, SUNLU). The origami bellows was 200 mm long with a maximum width of 78 mm. To maximize their extensibility and range of linear motion, the bellows were softened after printing in an 80 °C oven for 1 h while compressed to a length of 100 mm; they were then cooled to room temperature. Servo motor covers, motor horns, and bellows connectors were also 3D printed from TPU (TPU 95 A Black, SUNLU). For all the TPU 3D printed parts, the following print parameters were used: layer thickness of 0.2 mm, printing speeds from 50 to 300 mm s^−1^, 100% infill density, and printing temperature of 210 °C. The only non‐TPU 3D printed parts were the servo motor mounting plates and bellows‐HSA fasteners, which were 3D printed from polylactic acid (PLA, PLA Black, SUNLU). For the PLA parts, a layer thickness of 0.2 mm, printing speeds from 50 to 300 mm s^−1^, 50% infilled density, and a printing temperature of 220 °C were used. Based on estimates from Bambu Studio, the total materials cost for one actuator was $8.23 (USD): $7.85 of TPU and $0.38 of PLA. All parts were assembled and integrated with a servo motor (CLS3838MED, FLASH HOBBY).

### Servo Motor Actuation

Servo motors were operated with a 16‐channel, 12‐Bit PWM servo motor driver (PCA9685, HiLetgo) and Arduino Uno REV3 (ARD_A000066, Arduino). 8.4 V was supplied to operate servo motors at maximum rotation speed.

### Sensor Fabrication

Stretchable Kirigami sensors were constructed by sandwiching a conductive Kirigami layer between two non‐conductive Kirigami layers (See Figure S13, Supporting Information). The non‐conductive layers were 3D printed with a length of 100 mm, a width of 40 mm, and a thickness of 0.5 mm from TPU (TPU 95A Black, SUNLU). Conductive Kirigami layers were printed with the same dimensions using a conductive TPU filament (Conductive TPU Black, YOUSU). The sensor was assembled by attaching two wires to the conductive Kirigami layer; a heat‐adhesive sheet and heat press were used to assemble the full sensor.

### Data Acquisition

For all characterization experiments, the blocked force and torque data of the TPU HSA shaft, origami bellows, and assembled actuator were measured three times at a sample rate of ≈10–20 Hz using a 6‐axis load cell (RFT60‐HA01, ROBOTOUS). The displacement and coordinate data of the mechanical components, actuators, and full artificial musculoskeletal system were obtained by tracking color markers placed on the structures using a video analysis and modeling tool (Tracker, comPADRE). The Kirigami sensor's voltage response was measured using a 16‐bit analog‐to‐digital converter (ADS1115, Adafruit). Its programmable amplifying rate was set to measure a maximum voltage of 2.048 V.

### Axial and Bending Stiffness Characterization

To characterize the axial stiffness of the actuator, compression tests were performed by applying repeated axial compressions of 10 mm at the distal end of the actuator. The actuator was compressed five times at a constant speed of 5 mm s^−1^, across seven distinct actuator extension states, obtained by incrementally increasing the servo input angle from 0° to 270° in 45° steps. Compressive force data were recorded using a load cell positioned at the actuator's base. To assess the actuator's bending stiffness, the distal end of the actuator was displaced 20 mm five times at a speed of 5 mm s^−1^ as resulting tangential forces were measured using a load cell at the opposite, proximal end. Seven trials were conducted for seven initial actuator extensions (i.e., for servo input angles ranging from 0° to 270° in 45° increments).

### Calculation of Energy and Power Densities

The energy density (J kg^−1^) was calculated based on the maximum displacement (mm) values produced by the actuator in pushing or pulling motions under each load (kg) condition. Power density (W kg^−1^) was calculated based on the maximum extension and contraction speeds (m s^−1^) observed when the actuator operated under each load (kg).

### Artificial Musculoskeletal System

A robotic leg embodying an artificial musculoskeletal system was constructed from three architected soft actuators, three tendons, one femur, one shank, and foot‐like linkages that were joined using three linear guides and connecting brackets. The tendons for each actuator were 3D printed from TPU (TPU 95A Black, SUNLU). The femur and shank‐like linkages, foot structure, and connecting brackets were 3D printed from PLA (PLA Black, SUNLU). Three mini linear rail guides (MGN12H) were used to direct the linear motion of actuators. For the cyclic knee motion test, a battery (Zeee Premium Series 2S Lipo Battery, Zeee Power) with a nominal voltage of 7.4 V and a capacity of 8200 mAh was used; at 99% charged, it had a voltage of 8.37 V. The quad and hamstring actuators were operated using a servo motor driver (PCA9685, HiLetgo) connected to Arduino Uno REV3 (ARD_A000066, Arduino). Figure S14 (Supporting Information) shows the cyclic knee motion test setup.

## Conflict of Interest

A US patent has been filed by Northwestern University on this research.

## Supporting information



Supporting Information

Supporting Information

Supporting Information

Supporting Information

Supporting Information

Supporting Information

Supporting Information

Supporting Information

Supporting Information

Supporting Information

Supporting Information

Supporting Information

Supporting Information

Supporting Information

Supporting Information

Supporting Information

Supporting Information

Supporting Information

## Data Availability

The data that support the findings of this study are available from the corresponding author upon reasonable request.
