# Supplementary material for: Architected Soft Actuators for Artificial Musculoskeletal Systems
Source: Adv Mater. 2025 Jul 24;37(43):e01290. doi: 10.1002/adma.202501290 (PMC12574648; doi:10.1002/adma.202501290)
Supplement: Supplementary file 1 — Supporting Information [file ADMA-37-e01290-s006.pdf]

ADVANCED MATERIALS

Supporting Information

for *Adv. Mater.*, DOI 10.1002/adma.202501290

Architected Soft Actuators for Artificial Musculoskeletal Systems

*Taekyoung Kim, Eliot A. Dunn, Melinda Chen and Ryan L. Truby**

Supporting Information

Architected Soft Actuators for Artificial Musculoskeletal Systems

Taekyoung Kim[1,2], Eliot A. Dunn[1], Melinda Chen[1], and Ryan L. Truby[1,2,3,]*

[1]Department of Materials Science and Engineering, Northwestern University, Evanston, 60208, United States

[2]Center for Robotics and Biosystems, Northwestern University, Evanston, 60208, United States

[3]Department of Mechanical Engineering, Northwestern University, Evanston, 60208, United States

E-mail: rtruby@northwestern.edu

The file includes:

Supporting Notes

Note S1. Mathematical Model for Knee Motion of Artificial Robotic Leg.

Note S2. Influence of Reinforcing In-Fills on HSA Performance.

Supporting Tables

Table S1. Comparison of soft actuators.

Table S2. Comparison of soft actuators with mammalian skeletal muscle.

Table S3. Comparison of soft actuator power and energy densities using nominal total weight.

Supporting Figures

Figure S1. Actuator Fabrication Process.

Figure S2. Bellows Tensile Test at Varying Strain Rates.

Figure S3. Tensile and Torsional Stiffness Test.

Figure S4. Pulling Force of HSA Shaft.

Figure S5. Actuator Compressive Stress.

Figure S6. Actuator Tangential Force.

Figure S7. Reliability Test Results for Displacement, Pushing Force, and Pulling Force at 2 Hz and 5 Hz Actuation Frequencies.

Figure S8. Minimum Actuation Efficiency.

Figure S9. Impact of Linear Guide Rail on Knee Bending Motion.

Figure S10. Estimating Knee Motion.

Figure S11. Comparison of Peak Power Densities of Recent Soft Actuators.

Figure S12. Influence of In-filling Reinforcements on HSA

Figure S13. Kirigami Stretch Sensor Fabrication Process.

Figure S14. Cyclic Knee Motion Test Setup.

Supporting Movies

Movie S1 (.mp4 format). HSA Shaft Characterization – Blocked Pushing Force.

Movie S2 (.mp4 format). HSA Shaft Characterization – Free Displacement.

Movie S3 (.mp4 format). Origami Bellows Characterization – Tensile Testing.

Movie S4 (.mp4 format). Origami Bellows Characterization – Torsional Deflection.

Movie S5 (.mp4 format). Architected Soft Actuator Characterization – Free Displacement.

Movie S6 (.mp4 format). Architected Soft Actuator Characterization – Blocked Pushing Force.

Movie S7 (.mp4 format). Architected Soft Actuator Characterization – Blocked Pulling Force.

Movie S8 (.mp4 format). HSA Shaft Characterization – Blocked Pulling Force.

Movie S9 (.mp4 format). Architected Soft Actuator Performance – Cyclic Free Displacement.

Movie S10 (.mp4 format). Architected Soft Actuator Performance – Pushing and Pulling Varying Loads.

Movie S11 (.mp4 format). Architected Soft Actuator Performance – Durability Test with Applied Load.

Movie S12 (.mp4 format). Artificial Musculoskeletal System – Range of Motion Test.

Movie S13 (.mp4 format). Artificial Musculoskeletal System – System Response.

Movie S14 (.mp4 format). Artificial Musculoskeletal System – Pushing Force Test.

Movie S15 (.mp4 format). Artificial Musculoskeletal System – Ball Kicking Demonstration.

Movie S16 (.mp4 format). Artificial Musculoskeletal System – Proprioceptive Sensing.

Movie S17 (.mp4 format). Artificial Musculoskeletal System – Cyclic Knee Motion with a Battery.

Note S1. Mathematical Model for Knee Motion of Artificial Robotic Leg.

The combined mass of the calf actuator, connected linear guide and block, shank link, and foot structure located below the knee joint of the artificial robotic leg is approximately 0.95 kg. We assume the center of gravity for the lower part of the leg to be located about 445 mm from the knee joint. Based on this assumption, a torque of approximately 4.24 Nm is required to rotate the lower leg segment about the knee joint axis. This torque must be generated by the antagonistic actuation of the two proposed actuators mounted on the proximal upper leg.

Each actuator transmits force to the lower leg structure via a TPU tendon structure that applies the load at a point 62.5 mm from the knee joint axis. Consequently, to generate the required torque, each actuator must exert a force of approximately 34 N, which is equivalent to supporting a load of roughly 3.5 kg. Based on our characterization of the actuators, we know their pushing and pulling force output under 3.39 kg loads over a servo operating range of 0° to 270°. We can consider the antagonistic operation of the two actuators in the artificial leg – specifically, the actuators' displacement under a 3.39 kg load – and fit a fourth-order polynomial that maps servo rotation θ, to knee joint rotation, $f(\theta)$:

$$f(\theta) = 1.3 \times 10^{-8} \times \theta^4 - 8.3 \times 10^{-6} \times \theta^3 + 1.44 * 10^{-3} \times \theta^2 - 0.05883 \times \theta - 0.24844$$

Figure S10a presents a comparison between the predicted and actual knee joint angles as a function of servo angle input based on the extracted polynomial model. While the model was derived under slightly different load conditions, it successfully captures the general trend of the actual knee joint motion. When this joint angle data is converted into a trajectory in the X-Y plane, as illustrated in Figure S10b, both the predicted and actual trajectories exhibit similar overall shapes due to identical link lengths, with the maximum knee joint angle predicted by the model approximately 6° greater than the actual measured value. The difference between predicted and measured joint angle is likely due to any changes in soft actuator performance during leg operation (e.g., due to its mechanical compliance or changes during

operation from ideal linear extension) and interactions between leg components (e.g., at tendons, links, or the linear guides).

Note S2. Influence of Reinforcing In-Fills on HSA Performance.

We designed a reinforced HSA with thin wall structures added to the gaps between the auxetic patterns to generate increased pushing forces (see the inset as shown in Figure S12a). To verify the improved performance of the reinforced HSA, we assessed the blocked pushing forces generated by the HSA shaft with and without additional wall structures during actuation (Movie S1). During the loading and unloading phases, a similar trend with a hysteresis loop was observed in torque and pushing force outputs of HSAs with and without the wall structures due to the nonlinear, viscoelastic properties of TPU (Figure 2a and Figure S12b, Supporting Information). On the other hand, the pushing force and applied torque of the HSA with the walls were approximately 42% and 10% larger than those of the HSA without the walls, respectively. This result indicates that adding the wall structure to improve the HSA's performance was a reasonable solution because the increase in the pushing forces of the reinforced HSA was more significant than its increase in the applied torques.

Table S1. Comparison of soft actuators.

Actuator	Maximum Force Output	Maximum Strain	Actuation Frequency	Pros	Cons
This Work	High<br>(< 75 N)	Moderate<br>(30%)	Moderate to Fast<br>(1 Hz – 5 Hz)	- High force and peak power density<br>- Bidirectional actuation<br>- Simple to fabricate<br>- Low input voltage	- Motor required<br>- Limited design flexibility<br>- Complex material forms required
Pneumatic<br>(21)–(23), (38), (39)	High<br>(<100 N)	Large<br>(< 100% strain)	Moderate to Fast<br>(1 Hz – 10 Hz)	- High force & large strain<br>- Easy fabrication<br>- Design flexibility	- Bulky pumps, tanks, high pressures required<br>- Prone to failure<br>- Energy inefficient
DEA<br>(24)–(29),<br>(40) –(41)	Moderate<br>(<10 N)	Large<br>(< 300% strain)	Fast<br>(Hz – kHz)	- Fast, high actuation bandwidth<br>- Energy efficient<br>- High strain<br>- Silent	- High voltage<br>- Dielectric breakdown
SMA<br>(30)–(33),<br>(43) –(45)	Moderate to High<br>(<100 N)	Small<br>(< 8% strain)	Slow<br>(0.5 Hz – 2 Hz)	- High force and work capacity<br>- Silent	- Low actuation speed, bandwidth<br>- Energy inefficient<br>- Small strains
Tendon-driven<br>(34)–(37), (46)	High<br>(customizable)	Large<br>(customizable)	Moderate to Fast<br>(motor dependent; Hz)	- Precise & versatile<br>- High force transfer	- Complexity in cable routing<br>- Friction, mechanical wear, and backlash

Table S2. Comparison of soft actuators with mammalian skeletal muscle.

Type (Ref.)	Material Weight (kg)	Type of Power Source	Input Voltage (V)	Actuation Mode	Max Force(N)	Max Displaceme nt (mm) / Strain (%)	Peak Power Density (W/kg)	Peak Energy Density (J/kg)
Mammalian Skeletal Muscle (24)	-	-	-	Contraction	-	- / 40	200	40
This Work	0.32	Servo Motor	8.4 DC	Contraction	75	59 / 29.5	22.8	3.6
				Extension	74	59 / 29.5	12.3	3.7
Lattice Pneumatic Actuator (21)	0.08	Compressor	110 AC	Contraction	200	200 / 87.5	-	286
Origami Pneumatic Actuator (22)	0.003	Vacuum Pump	110 AC	Contraction	428	70 / 90	2000	-
Dielectric Elastomer Actuator (24)	0.02	High Voltage Amplifier	3750 DC	Contraction	70	- / 24	80	19.8
				Extension	-	- / 22	80	13.8
HASEL (26)	-	High Voltage Amplifier	8,000 to 20,000 DC	Extension	40	55 / 69	614	70
Peano HASEL (27)	0.005	High Voltage Amplifier	8,000 to 13,000 DC	Contraction	20	5.43 / 8	160	-
HALVE (29)	0.003	Voltage Amplifier	1,100 DC	Extension	16.3	- / 971	50.5	10.3
SEMA (61)	0.011	Low Voltage Supply	3-12 DC	Contraction<br>Extension	2.8	6 / -	5.3	0.4

Table S3. Comparison of soft actuator power and energy densities using nominal total weight.

Type (Ref.)	Material Weight (kg)	Power Source Weight (kg)	Nominal Total Weight (kg)	Actuation Mode	Peak Power Density (W/kg)	Peak Energy Density (J/kg)
This Work	0.32	0.08	0.4	Contraction	18.24	2.88
				Extension	9.84	2.96
Lattice Pneumatic Actuator (21)	0.08	38	38	Contraction	-	0.60
Origami Pneumatic Actuator (22)	0.003	11.2	11.2	Contraction	0.54	-
Dielectric Elastomer Actuator (24)	0.02	10.6	10.6	Contraction	0.15	0.04
				Extension	0.15	0.03
HASEL (26)	-	125	>125	Extension	-	-
Peano HASEL (27)	0.005	125	125	Contraction	0.01	-
HALVE (29)	0.003	0.5	0.5	Extension	0.303	0.06
SEMA (61)	0.011	0.5	0.5	Contraction<br>Extension	0.12	0.01

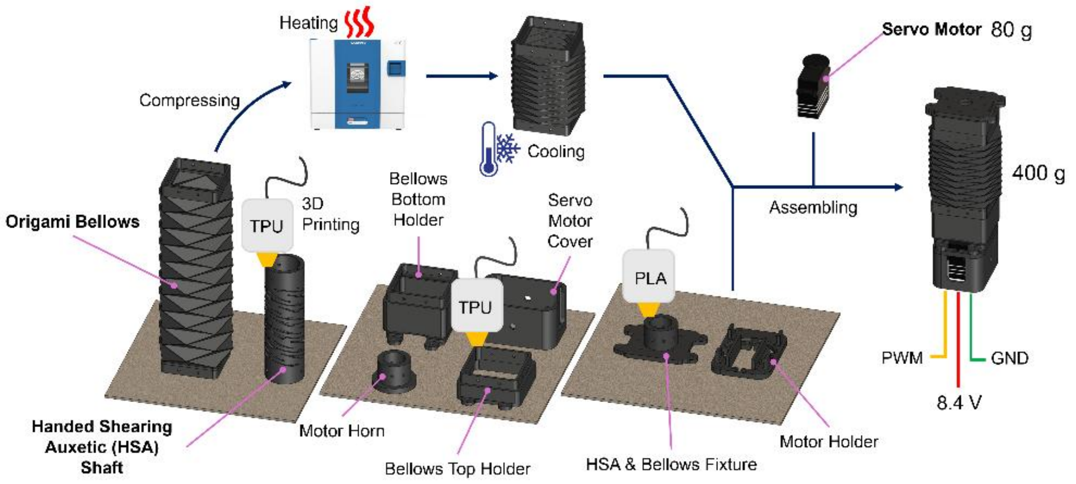

Figure S1. **Actuator Fabrication Process.** Except for a PLA servo motor mounting plate and a PLA bellows-HSA fixture, all actuator components are 3D printed from TPU. The printed Yoshimura origami bellows structure is heated in the oven and cooled to compress it before assembling with every prepared part, including a servo motor.

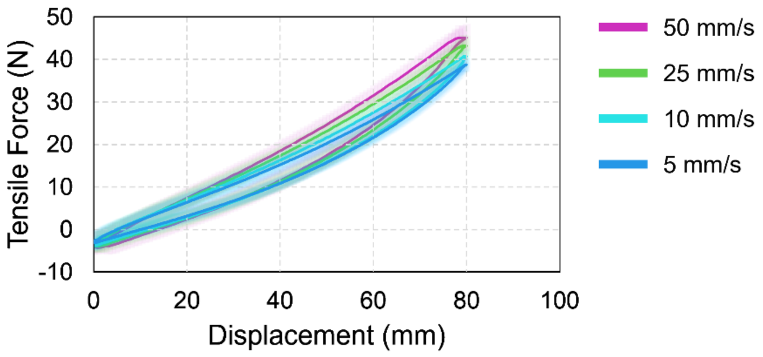

Figure S2. Bellows Tensile Test at Varying Strain Rates. Tensile force versus displacement at strain rates of 5, 10, 25 and 50 mm/s. Shaded error bands indicate standard deviation (n = 5).

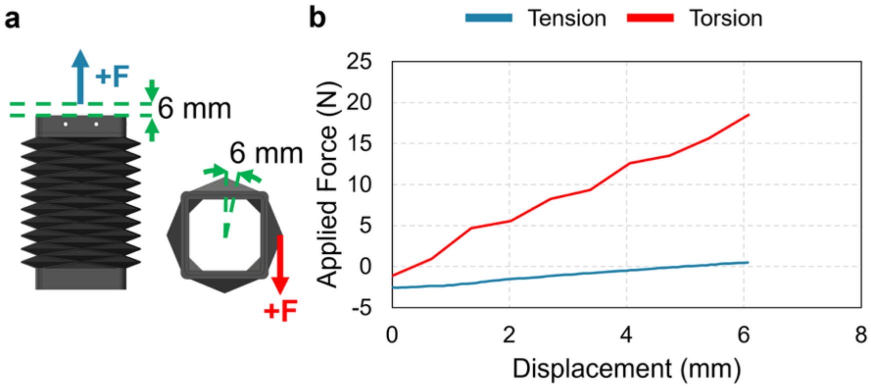

Figure S3. Tensile and Torsional Stiffness Test. (a) The origami bellows structure is extended and rotated 6 mm, which corresponds to the arc length of rotating the bellows 9˚, to evaluate differences in axial and torsional stiffness. (b) The force versus displacement response for each loading case in (a).

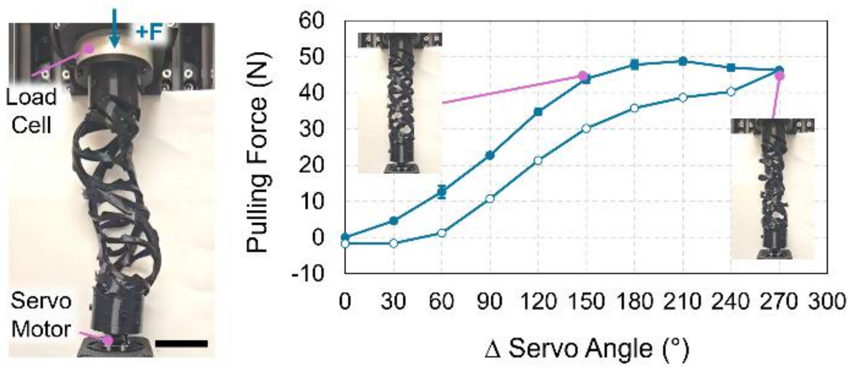

Figure S4. **Pulling Force of HSA Shaft.** The photograph at left shows the experimental setup used to measure the blocked pulling force, F, of a reinforced HSA as a function of servo rotation angle. The plot at right shows the pulling force versus servo rotation angle. Closed and open circles indicate loading and unloading data, respectively. Error bars indicate standard deviation (n = 3). Scale bar is 50 mm.

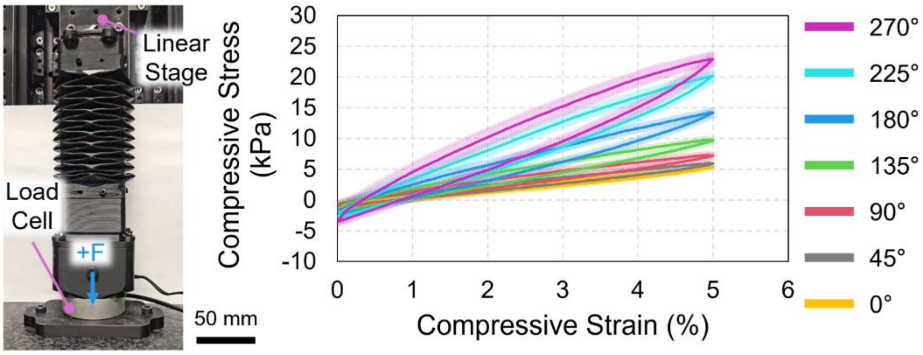

Figure S5. Actuator Stiffness Increases During Actuation. The photograph at left shows the experimental setup used to measure compressive forces, F, of the actuator using a load cell while pushing the distal end of the actuator in longitudinal direction. The plot on the right shows the compressive stress and strain during five loading and unloading cycles (5 mm/2) for the actuator at seven different initial lengths set by the servo rotation angle.

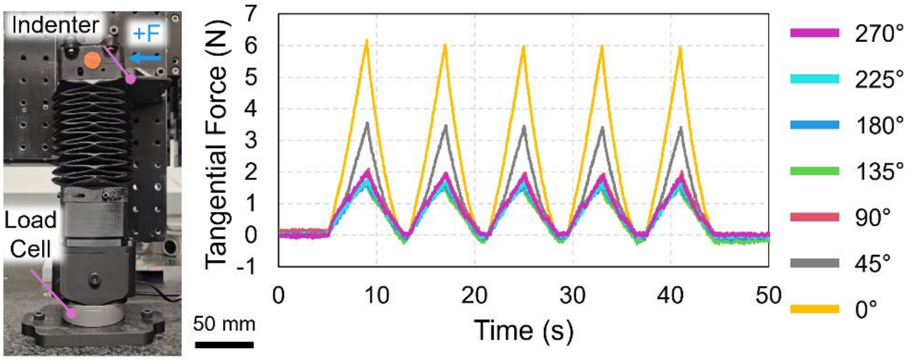

Figure S6. Actuator Tangential Force. The photograph at left shows the experimental setup used to measure the tangential force, F, on the actuator using a load cell while pushing the distal end of the actuator. The plot on the right shows the tangential force during five bending cycles of the actuators when actuated to several starting lengths using servo rotation angles of 0˚ to 270˚**.**

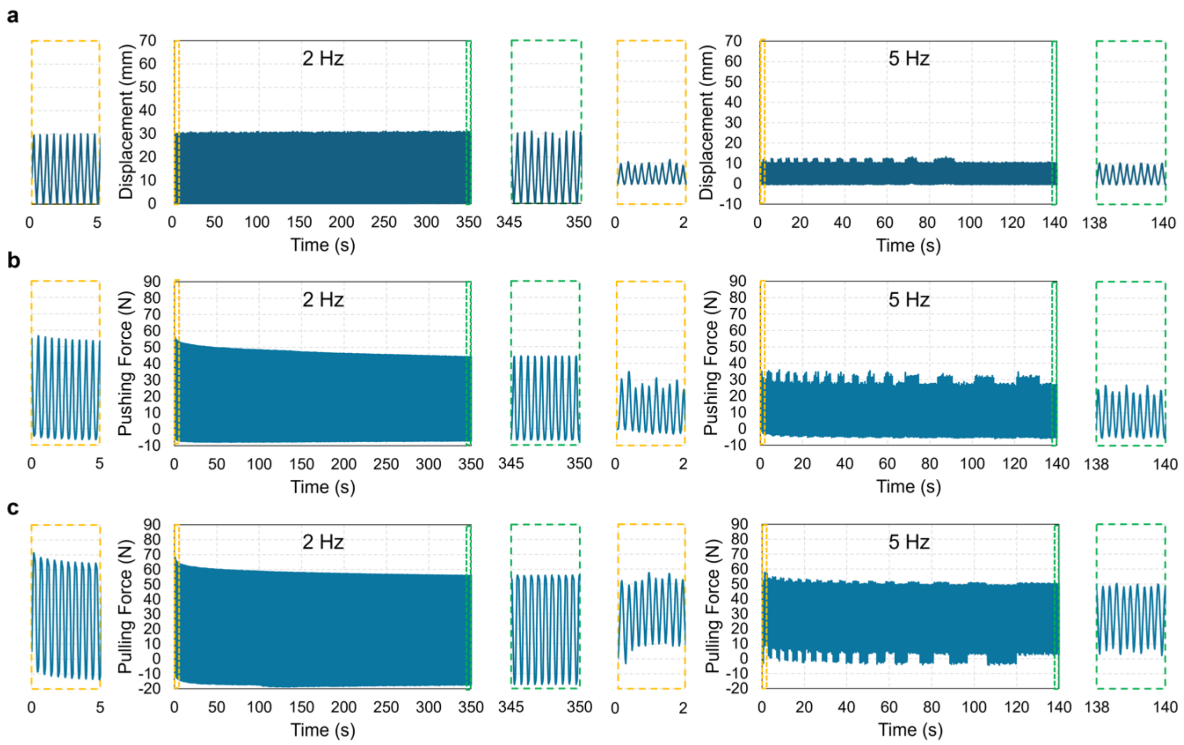

Figure S7. Reliability Test Results for Displacement, Pushing Force, and Pulling Force at 2 Hz and 5 Hz Actuation Frequencies. The plots in (a) to (c) show reliability test results for each actuation case at 2 Hz (left) and 5 Hz (right); (a) displacement, (b) pushing force, and (c) pulling force responses are provided over 700 actuation cycles. The yellow and green insets show results at the beginning and end of the reliability tests, respectively.

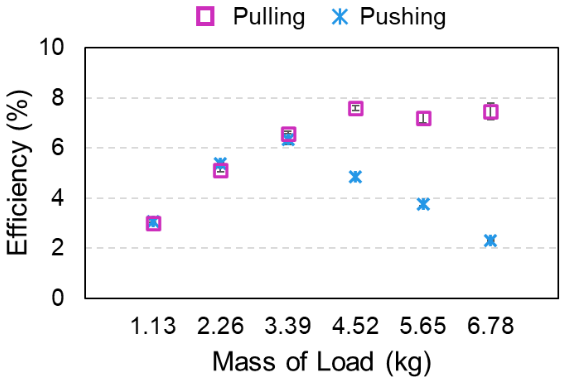

Figure S8. Minimum Actuation Efficiency. Energy efficiencies are provided for each pulled and pushed load shown in Figure 4. Error bars and shaded error bands indicate standard deviation (n = 5).

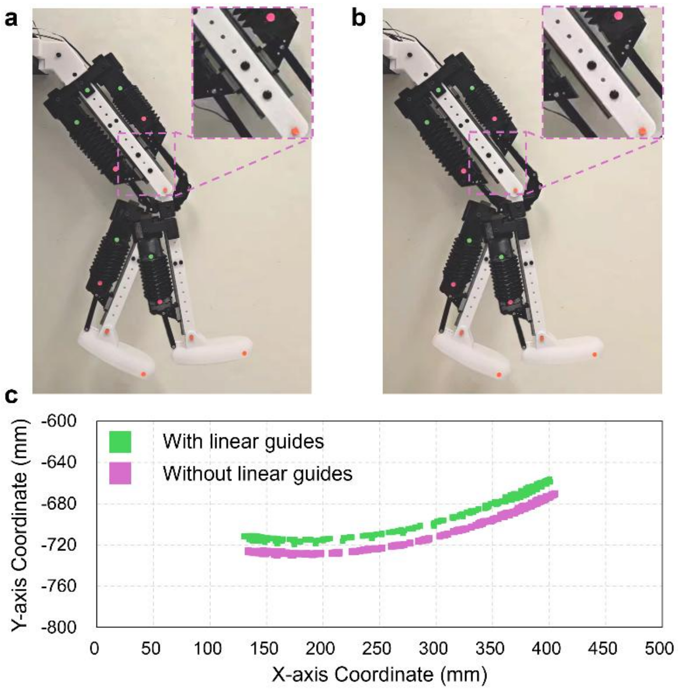

Figure S9. **Impact of Linear Guide Rail on Knee Bending Motion.** The overlaid photographs indicate the full range of knee motions for when the actuators are (a) connected (b) or disconnected to the linear rail guide. (c) The trajectories of the foot are shown for each case.

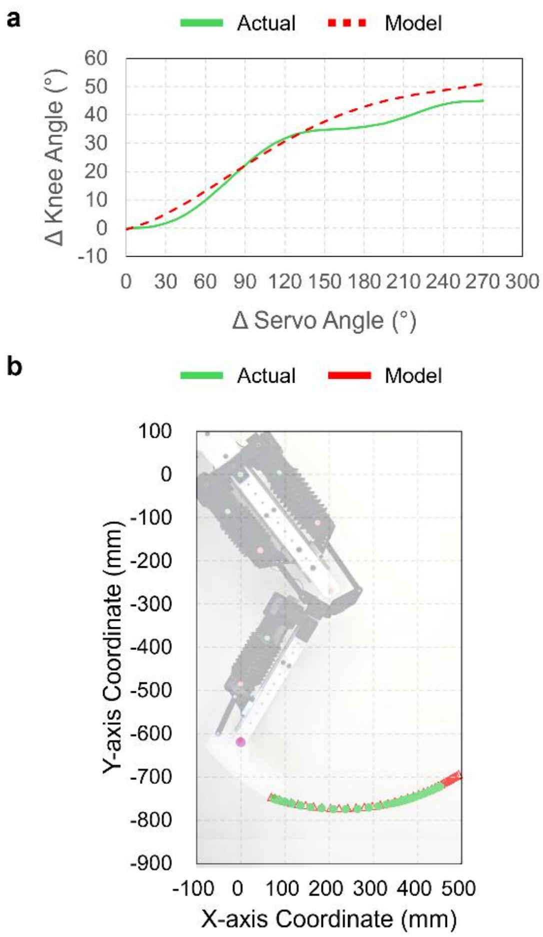

Figure S10. Estimating Knee Motion. (a) Comparison between the predicted and actual knee joint angles as a function of servo angle based on the extracted polynomial model. (b) Actual and model predicted trajectories of a marker on the foot structure in the X-Y plane are provided.

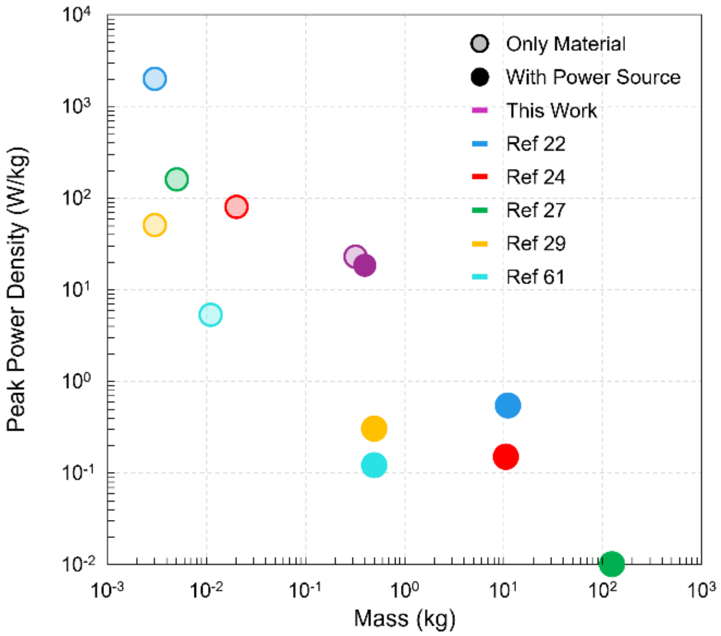

Figure S11. Comparing Peak Power Densities of State-of-the-Art Soft Actuators. The Ashby chart compares peak power density versus mass for several actuators. The lighter circles represent the peak power densities of actuators when only the material mass of the actuator is considered. Darker circles represent the peak power densities when the mass of the associated power sources (e.g., pumps, servo) is included in the overall mass of the actuator.

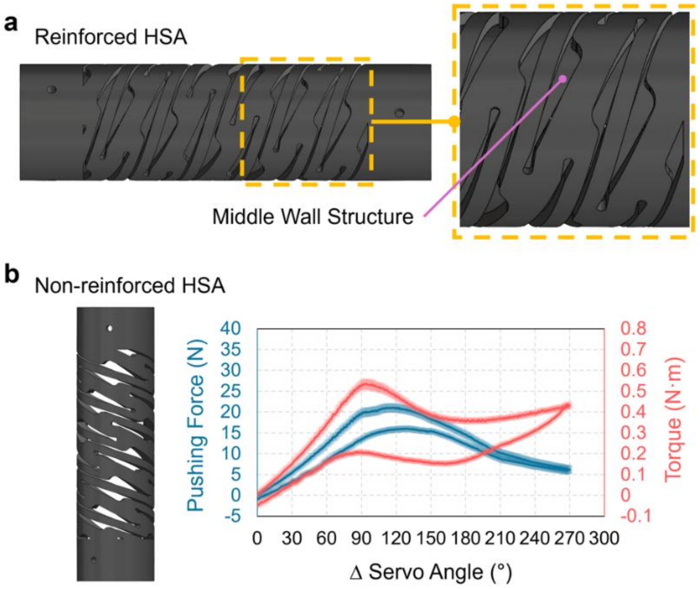

Figure S12. Influence of In-filling Reinforcements on HSA. (a) The CAD image of HSAs with middle wall structures. The inset shows a close-up image of the middle wall structure. (b) The CAD image of HSAs without middle wall structures (left). The characterization result of an HSA without middle wall structures—pushing force and torque of the HSA versus servo rotation angle—are shown. Shaded error bands indicate standard deviation (n = 3).

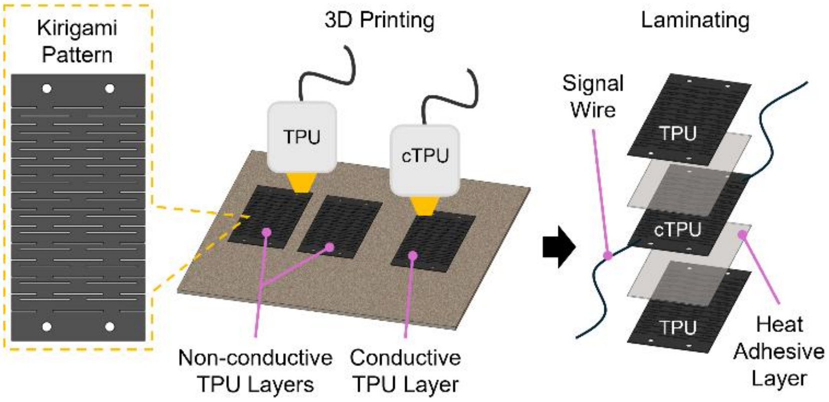

Figure S13. Kirigami Stretch Sensor Fabrication Process. Two non-conductive and one conductive TPU (cTPU) Kirigami layers are 3D printed using a FDM printer (left). The CAD model in the inset shows the printed stretchable Kirigami pattern. The prepared layers and signal wires are sandwiched using heat adhesive layers by heat pressing (right).

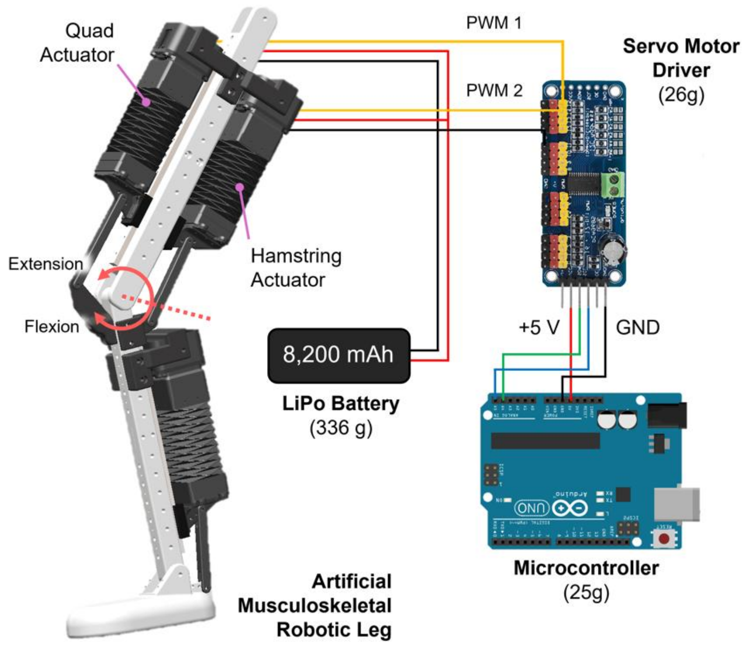

Figure S14. Cyclic Knee Motion Test Setup. Quad and hamstring actuators are powered by a LiPo battery and controlled by a servo motor driver connected to a microcontroller to generate knee motions.

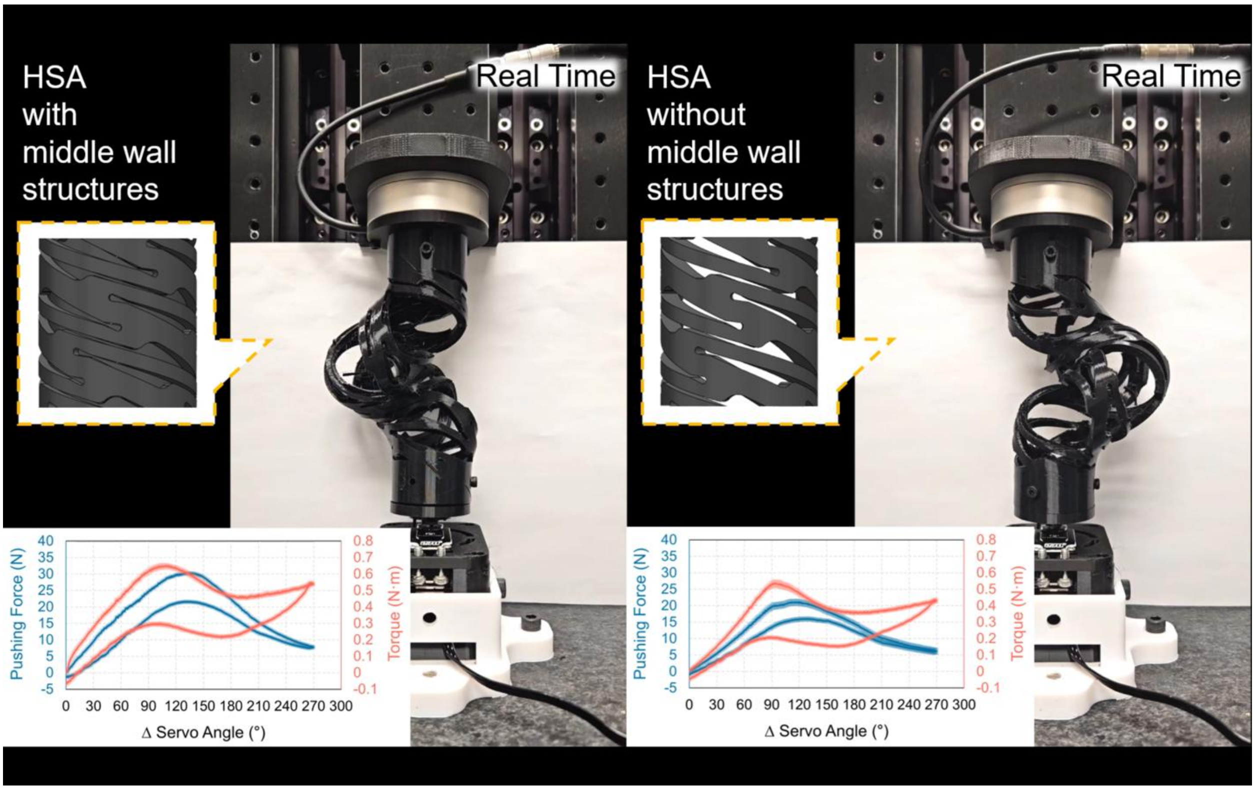

Movie S1 (.mp4 format). HSA Shaft Characterization – Blocked Pushing Force (real-time playback). The blocked pushing forces of HSAs with and without middle wall reinforced structures were measured using a load cell while servo motors rotated from 0° to 270° and returned to 0°. The corresponding torques were also measured at the same time.

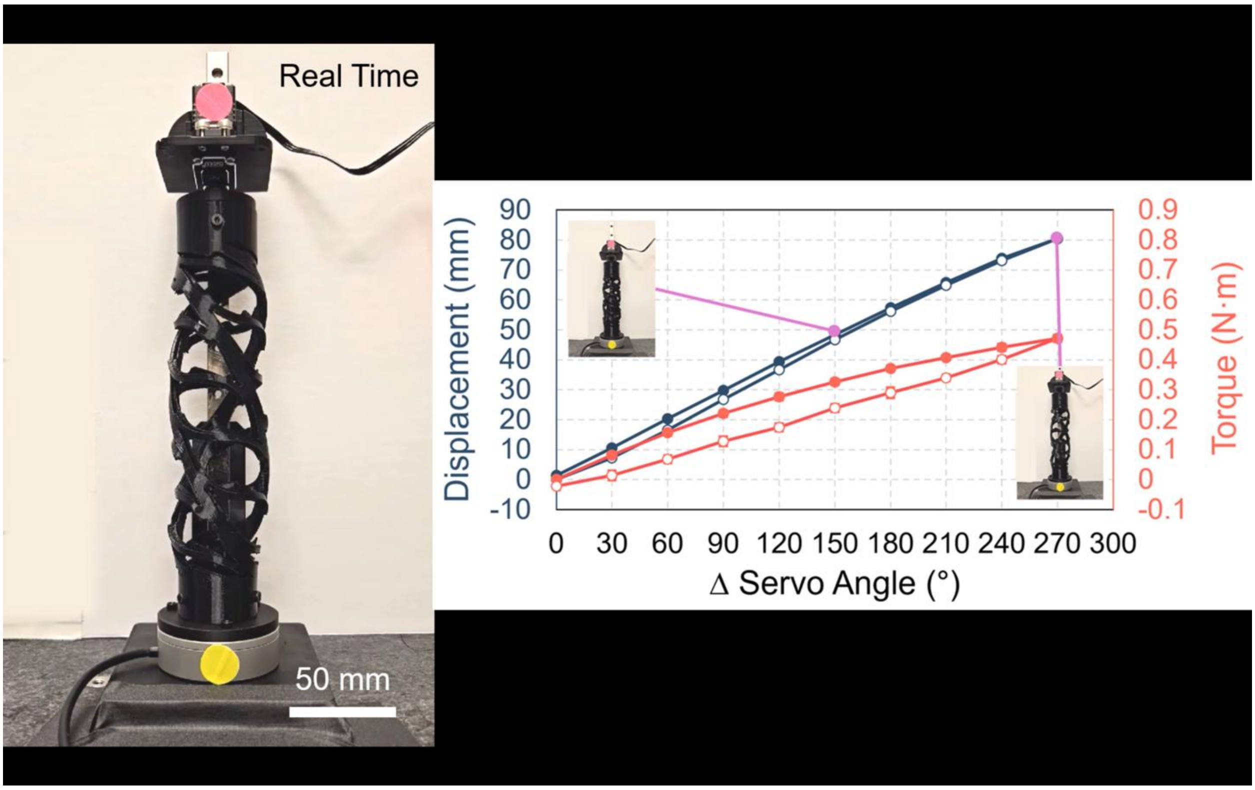

Movie S2 (.mp4 format). HSA Shaft Characterization – Free Displacement (real-time playback). The free displacement of an HSA was measured by tracking a pink color marker using image tracking software while servo motors rotated from 0° to 270° and back at 30° increments. The corresponding torques were measured using a load cell.

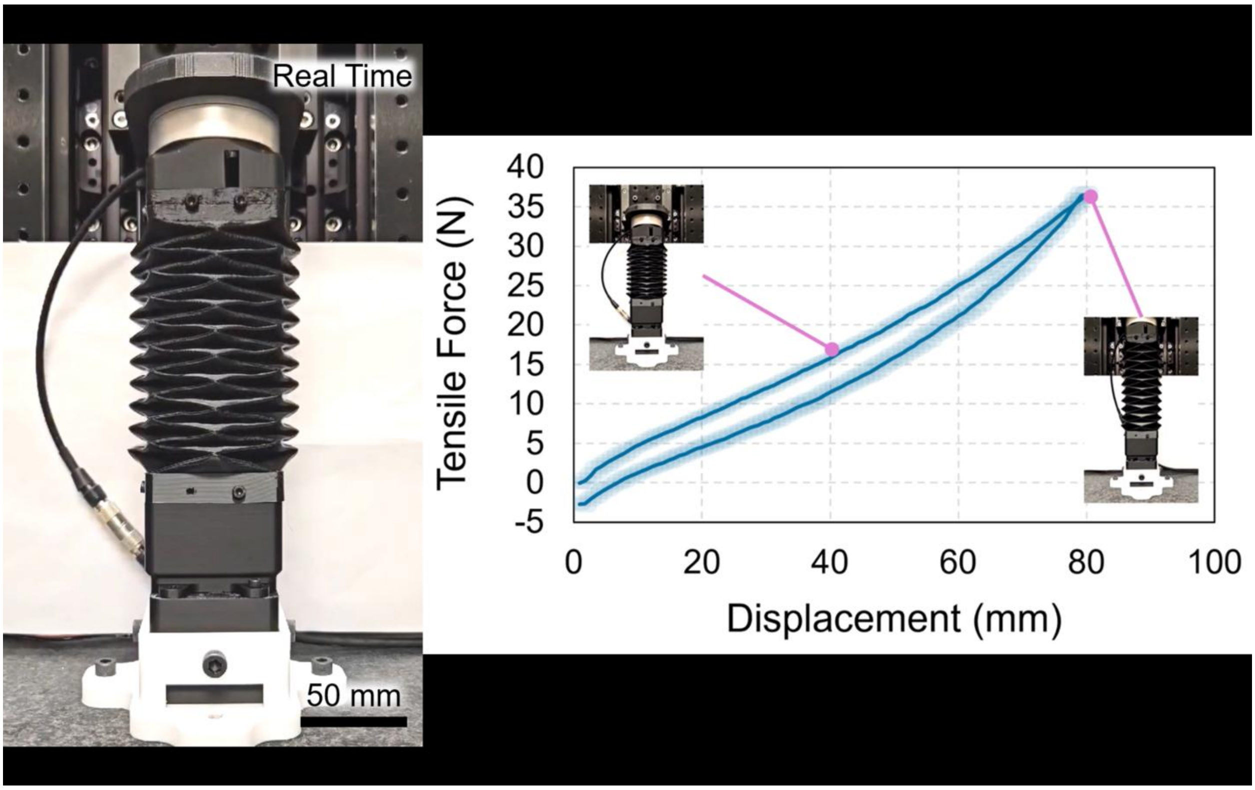

Movie S3 (.mp4 format). Origami Bellows Characterization – Tensile Testing (real-time playback). The tensile force of an origami bellows was measured by a load cell while a linear robotic stage stretched the bellows up to 80 mm and released it to its initial length.

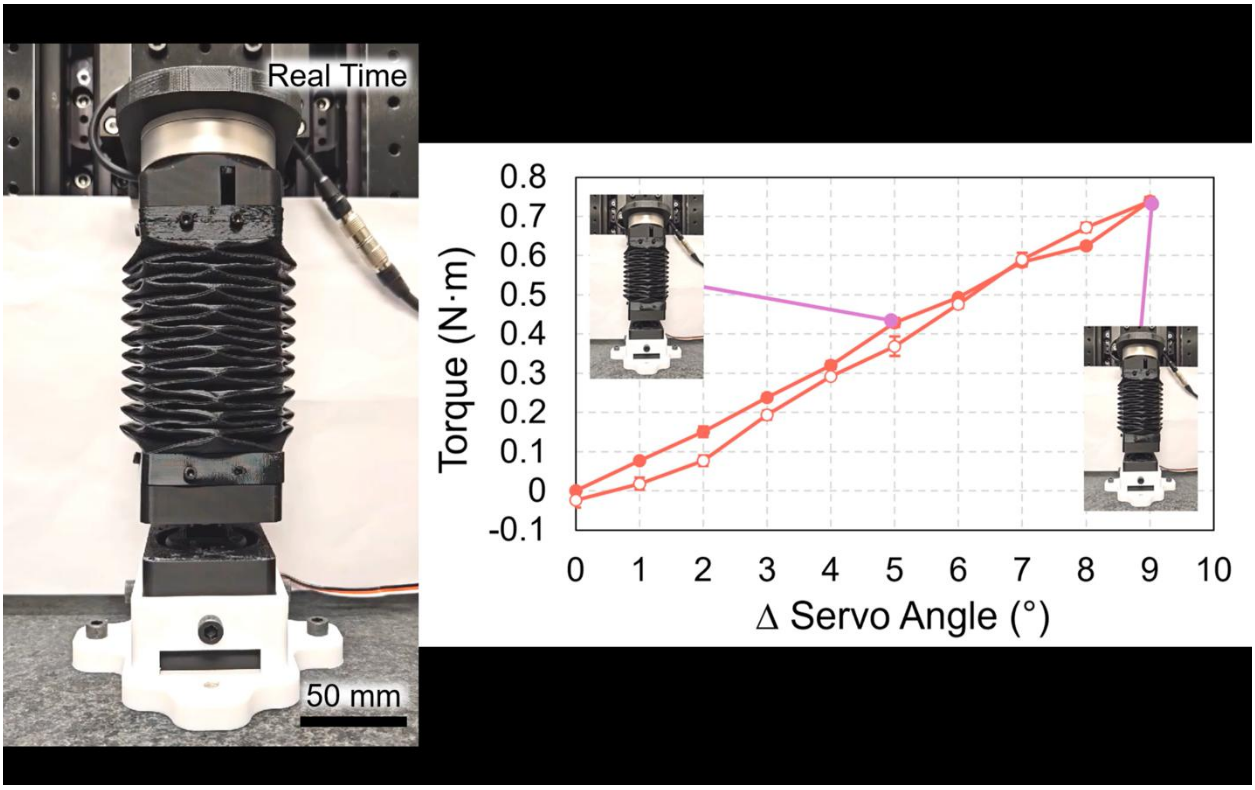

Movie S4 (.mp4 format). Origami Bellows Characterization – Torsional Deflection (real-time playback). The torsional deflection was applied to an origami bellows by rotating a servo motor from 0° to 9° and back at a 1° increment, while the applied torque was measured by a load cell.

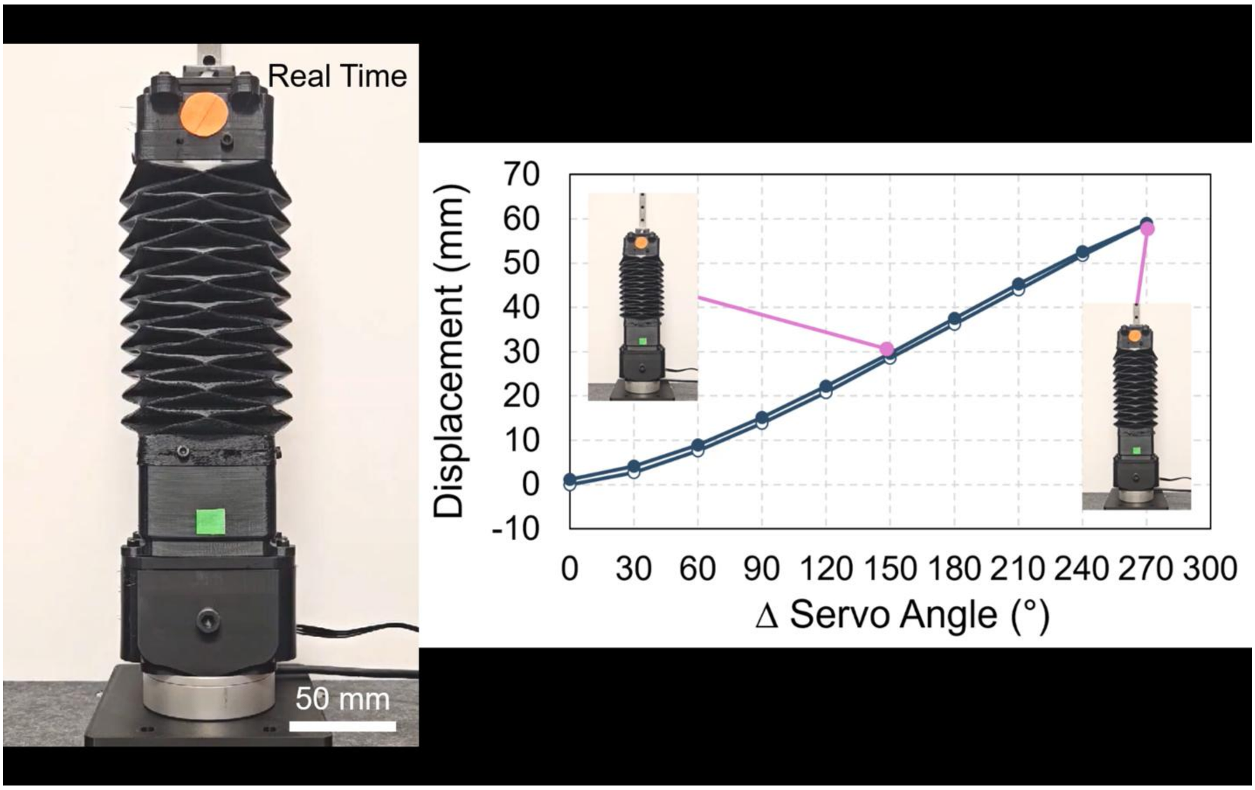

Movie S5 (.mp4 format). Architected Soft Actuator Characterization – Free Displacement (real-time playback). The free displacement of an assembled actuator was measured by tracking an orange color marker using image tracking software; servo motors were rotated from 0° to 270° and back at a 30° increment.

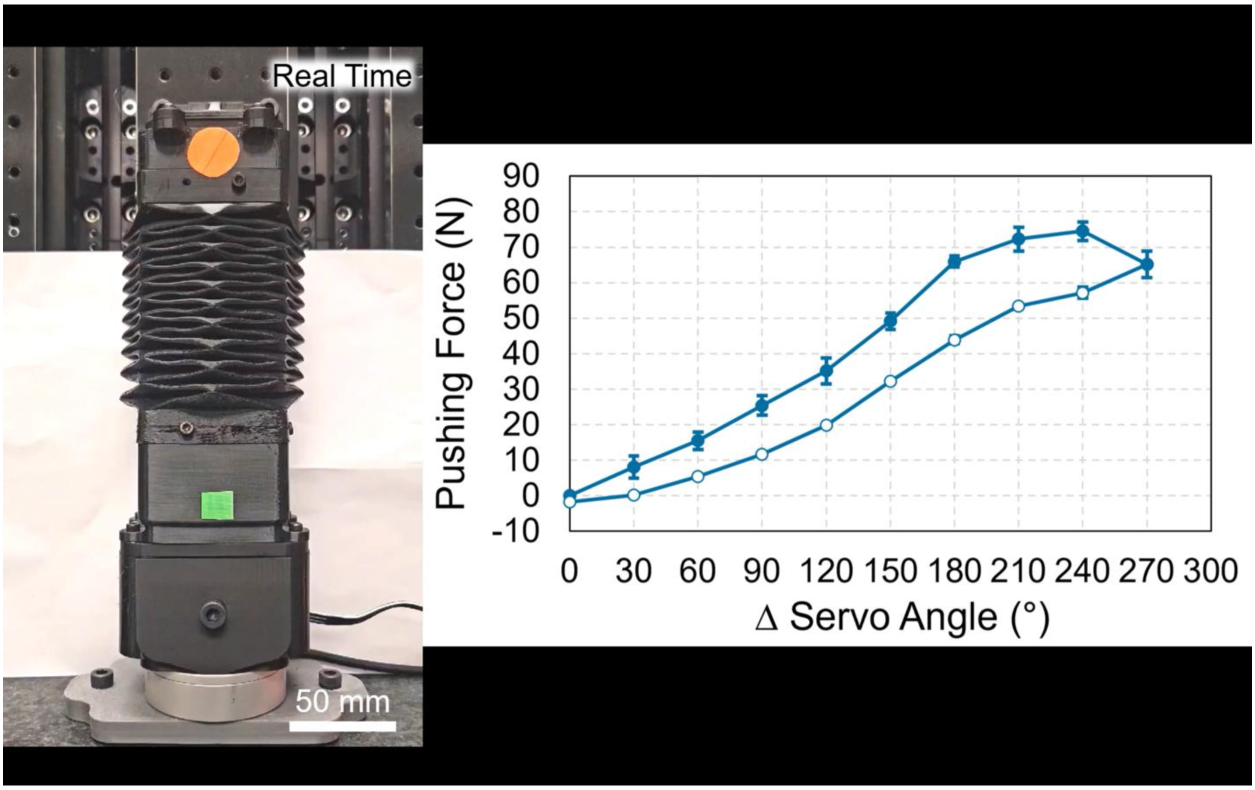

Movie S6 (.mp4 format). Architected Soft Actuator Characterization – Blocked Pushing Force (real-time playback). The blocked pushing force of an assembled actuator was measured using a load cell while servo motors rotated from 0° to 270° and back at a 30° increment.

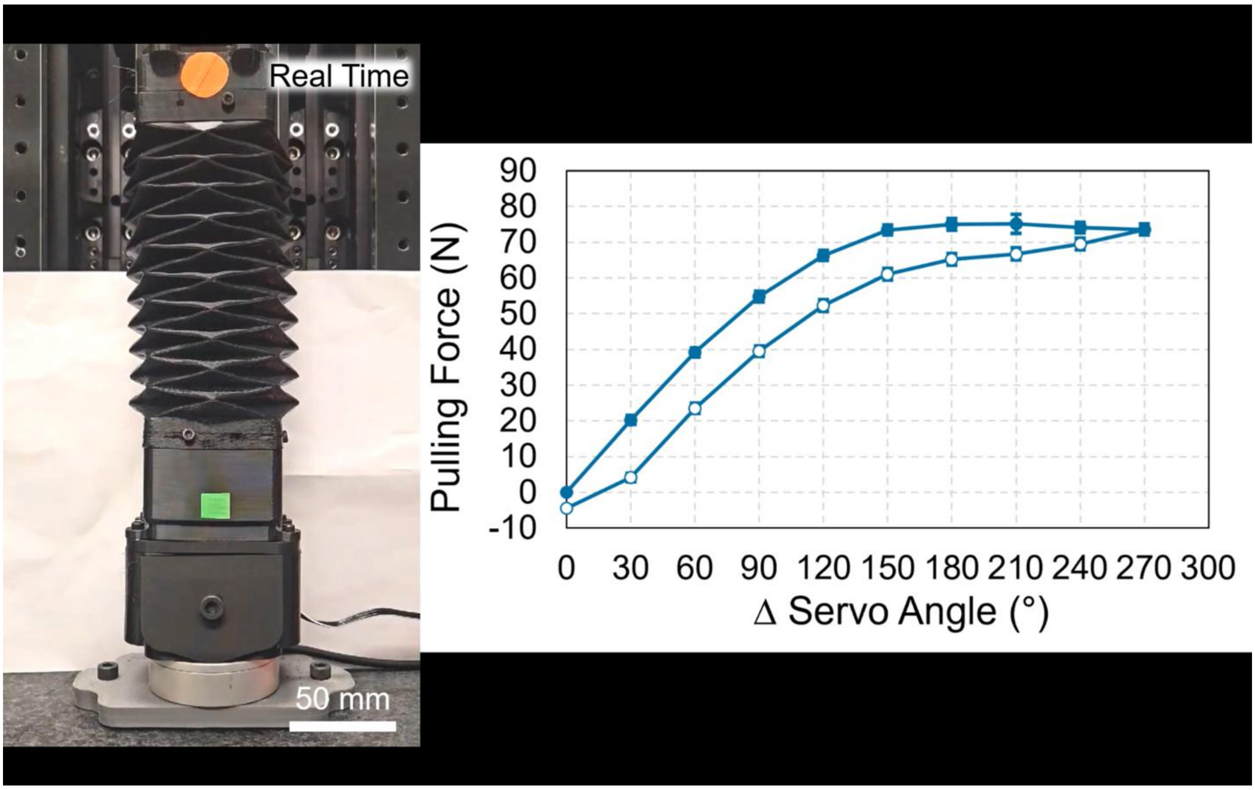

Movie S7 (.mp4 format). Architected Soft Actuator Characterization – Blocked Pulling Force (real-time playback). The blocked pulling force of an assembled actuator was measured using a load cell in the actuator's fully extended state while the motor was rotated down to 0° and back to 270° at a 30° increment.

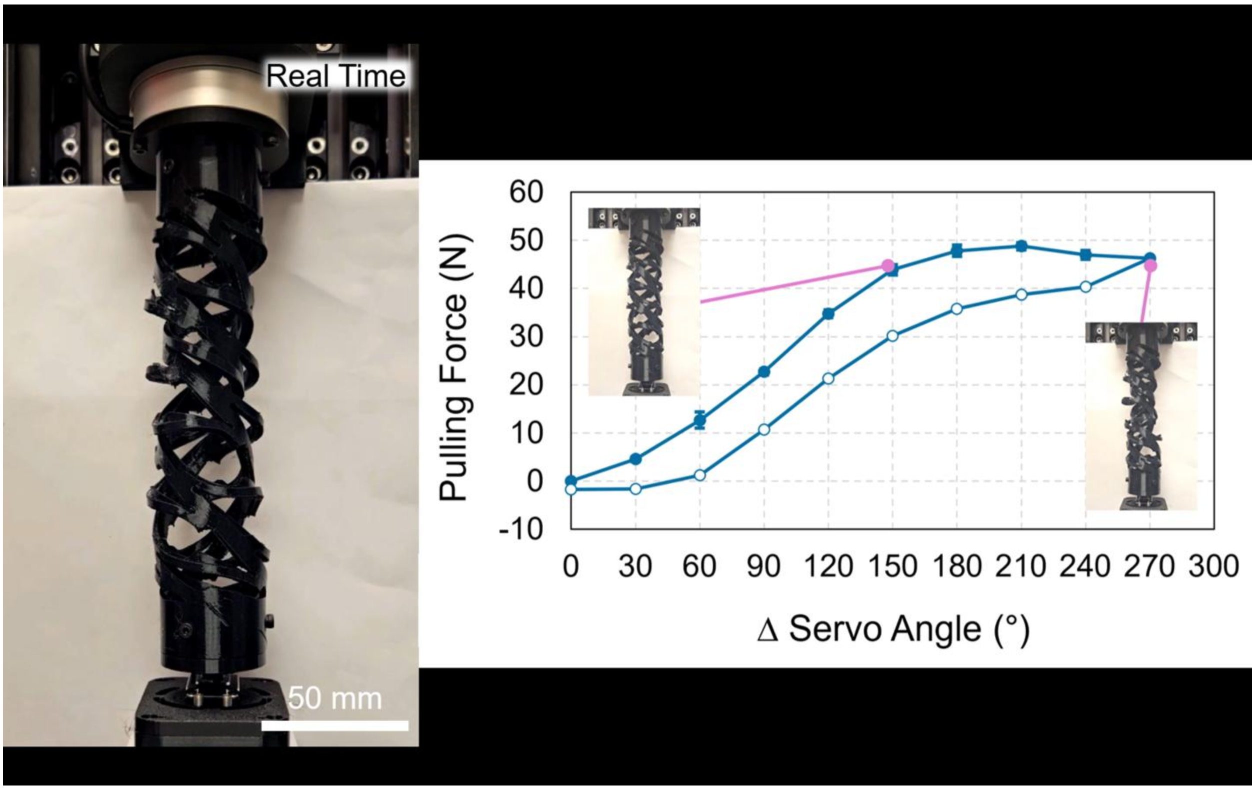

Movie S8 (.mp4 format). HSA Shaft Characterization – Blocked Pulling Force (real-time playback). The blocked pulling force of an HSA was measured using a load cell in the HSA's fully extended state while the motor was rotated down to 0° and back to 270° at a 30° increment.

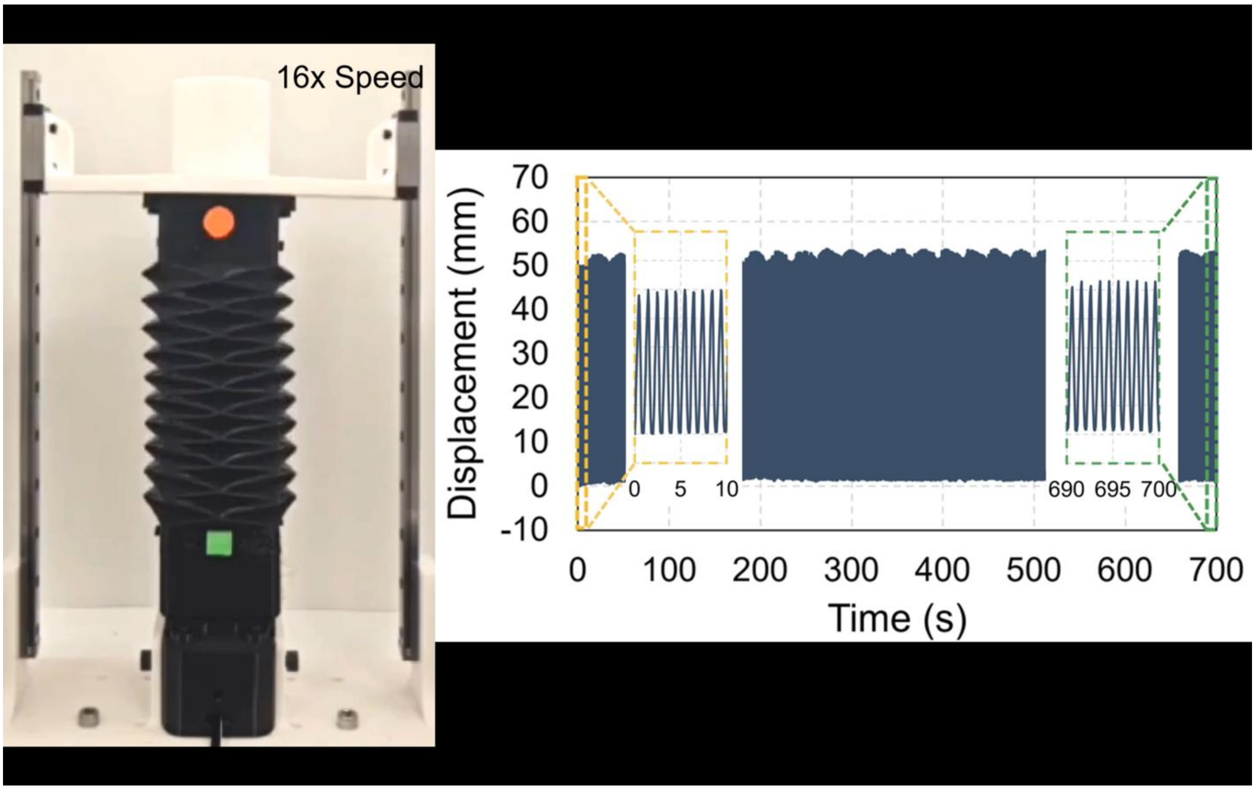

Movie S9 (.mp4 format). Architected Soft Actuator Performance – Cyclic Free Displacement (16× playback). Free displacement reliability tests were conducted by operating the servo motor between 0° to 270° rotation angles at 1-s periods to generate strokes at its maximum speed.

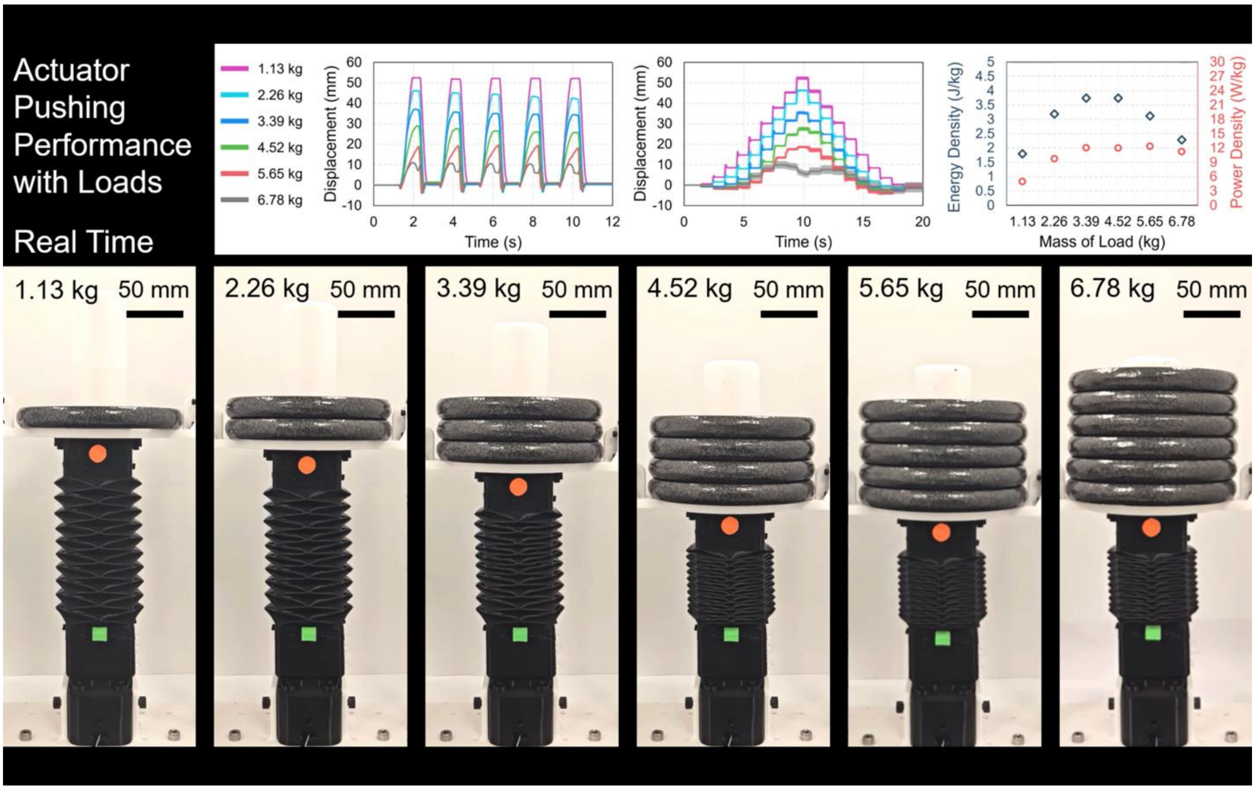

Movie S10 (.mp4 format). Architected Soft Actuator Performance – Pushing and Pulling Varying Loads (real-time playback). To lift the weights by pushing, the actuator was driven by operating the servo motor up to a 270° rotation angle at maximum speed and back to 0° while increasing the number of weights. The pulling tests were conducted by hanging the actuator upside down and loading the weights in increasing amounts, one by one, up to a total of six weights. The actuator was fully extended at 270° servo rotation angle to set the initial state, and the servo motor rotated down to 0° and back to the initial 270° at maximum speed.

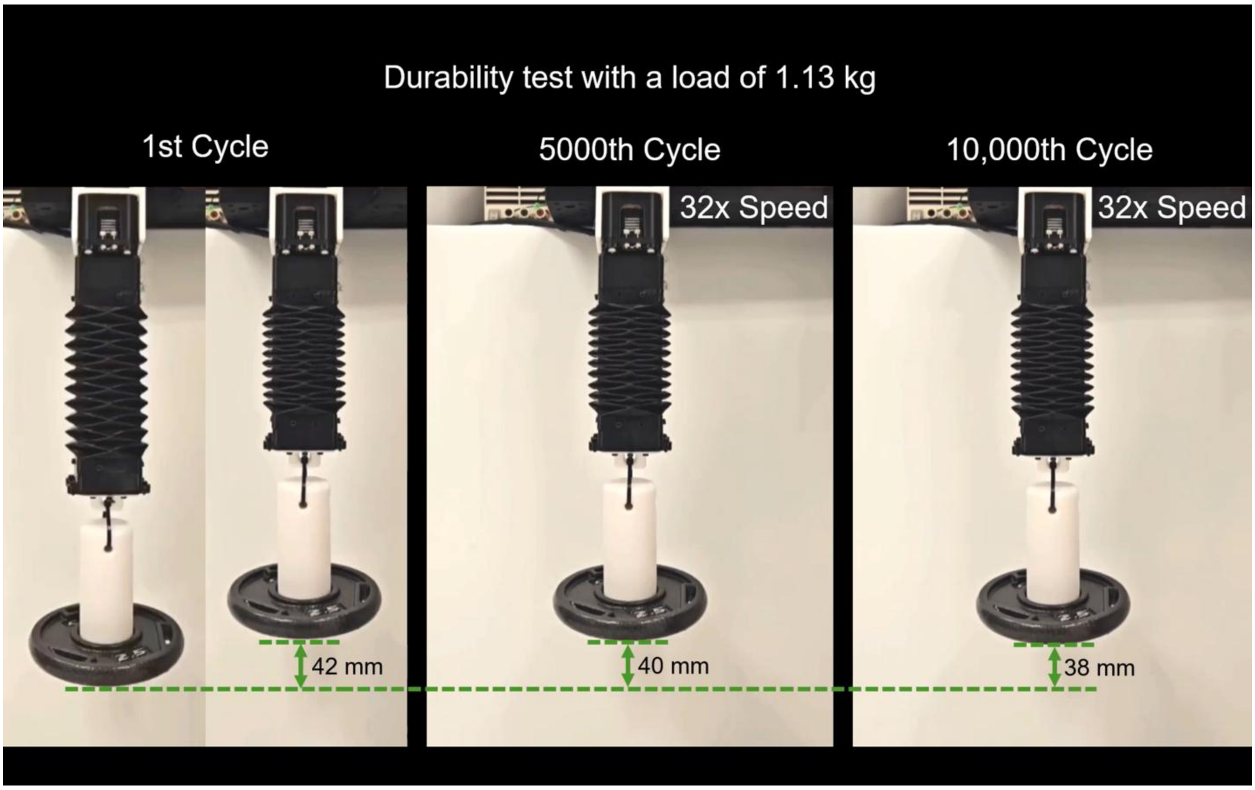

Movie S11 (.mp4 format). Architected Soft Actuator Performance – Durability Test with Applied Load (32× playback). A durability test was conducted for pulling a weight of 1.13 kg for 10,000 cycles at 1s period.

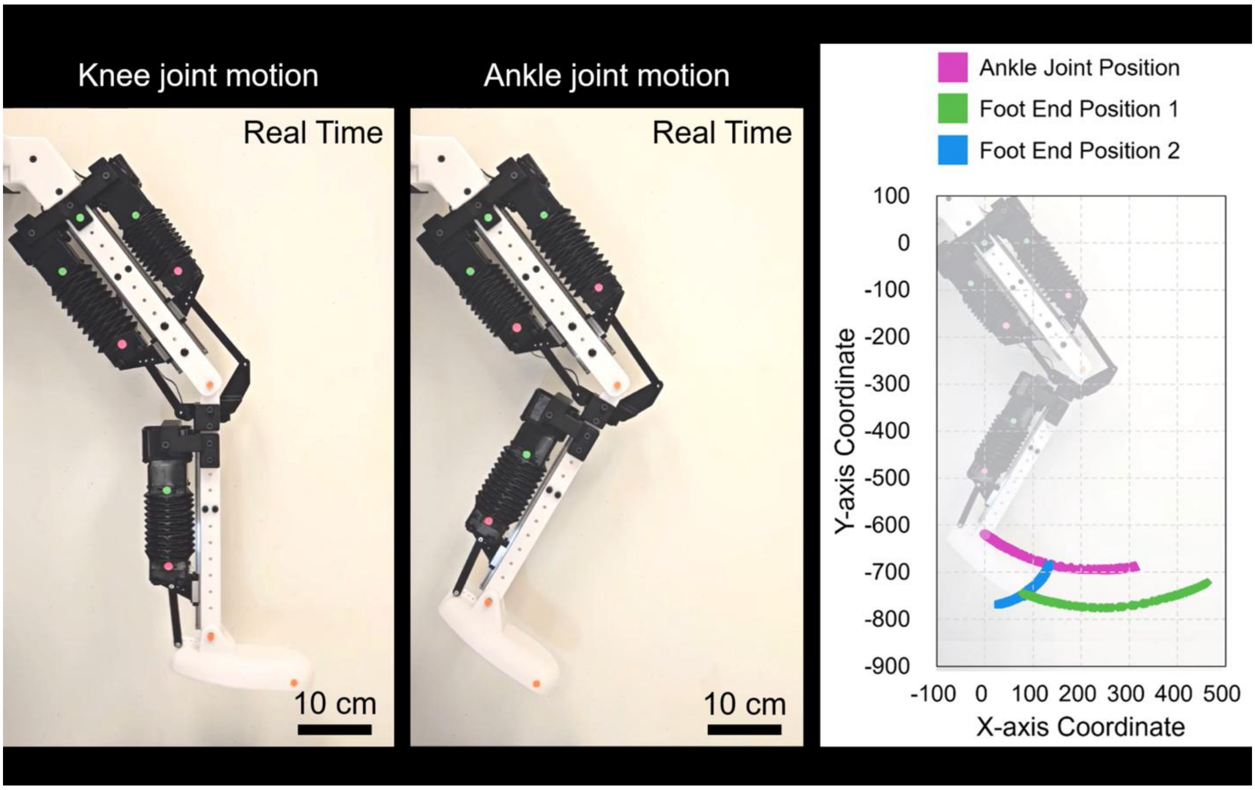

Movie S12 (.mp4 format). Artificial Musculoskeletal System – Range of Motion Test (real-time Playback). To characterize the range of motion for the knee, the quad and hamstring actuators were operated at their maximum speed for a 700-ms period to extend and contract, respectively, to their full extent. For evaluating ankle movement, the calf actuator repeatedly contracted and extended from the initial state where the servo angle was 135°.

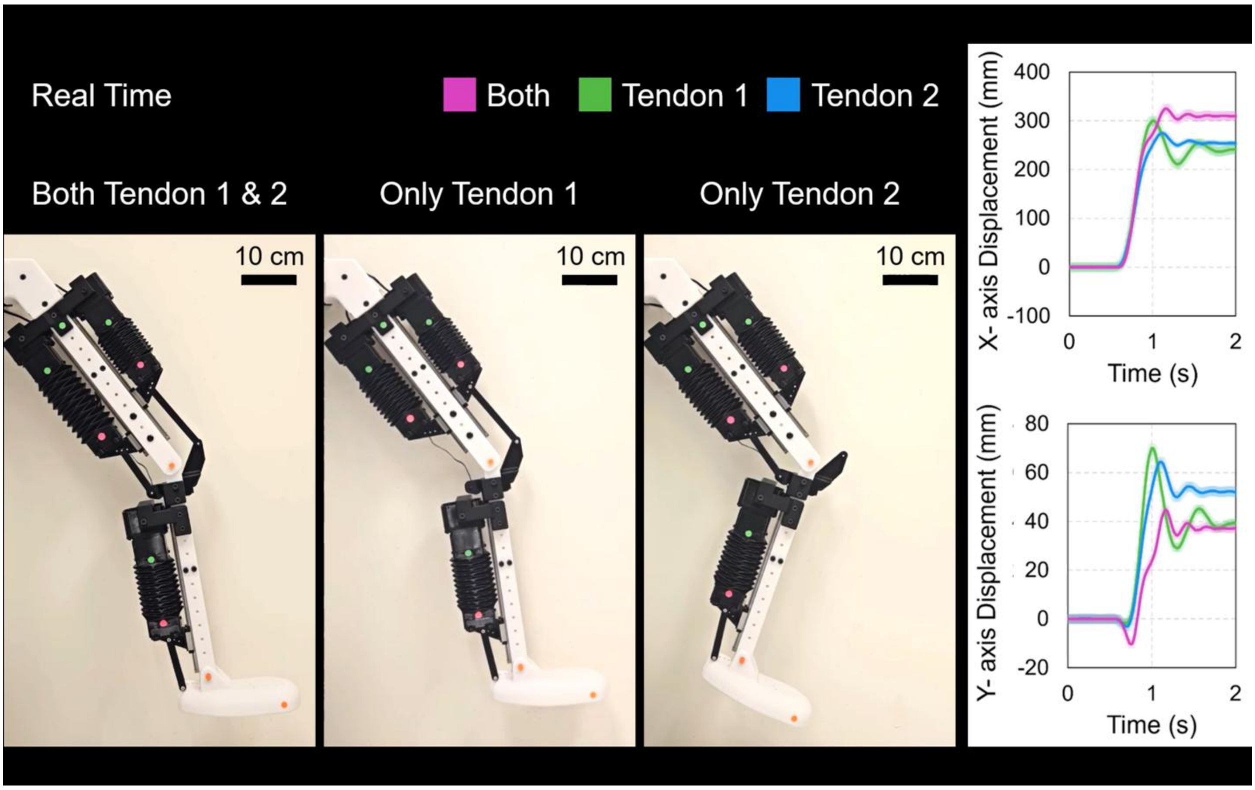

Movie S13 (.mp4 format). Artificial Musculoskeletal System – System Response (real-time playback). A full range of knee motions corresponding to a servo rotation range of 270° was performed for three different cases – using both quad and hamstring, only quad, and only hamstring – to demonstrate the mechanical response of the leg-inspired artificial musculoskeletal system.

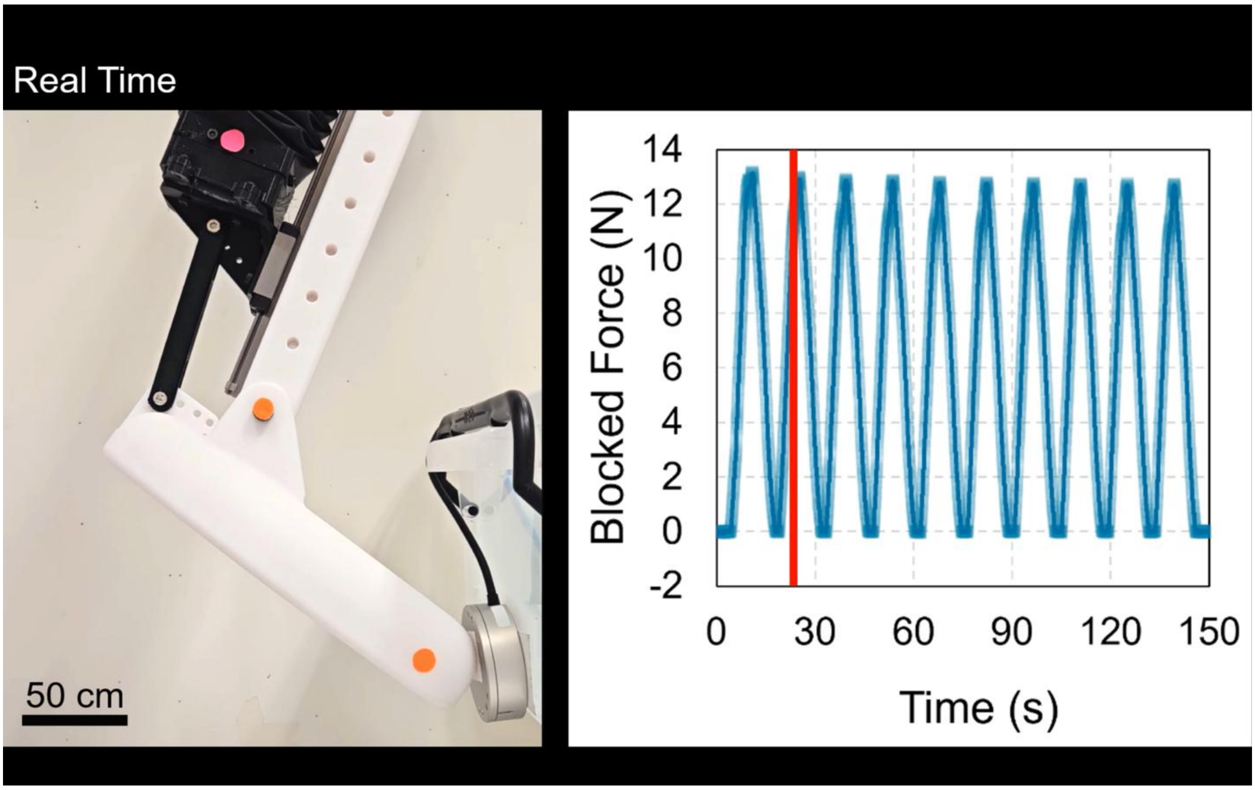

Movie S14 (.mp4 format). Artificial Musculoskeletal System – Pushing Force Test (real-time playback). Blocked pushing force at the toe was measured using a load cell as the quad and hamstring actuators were driven by their maximum servo rotation of 270° for ten cycles.

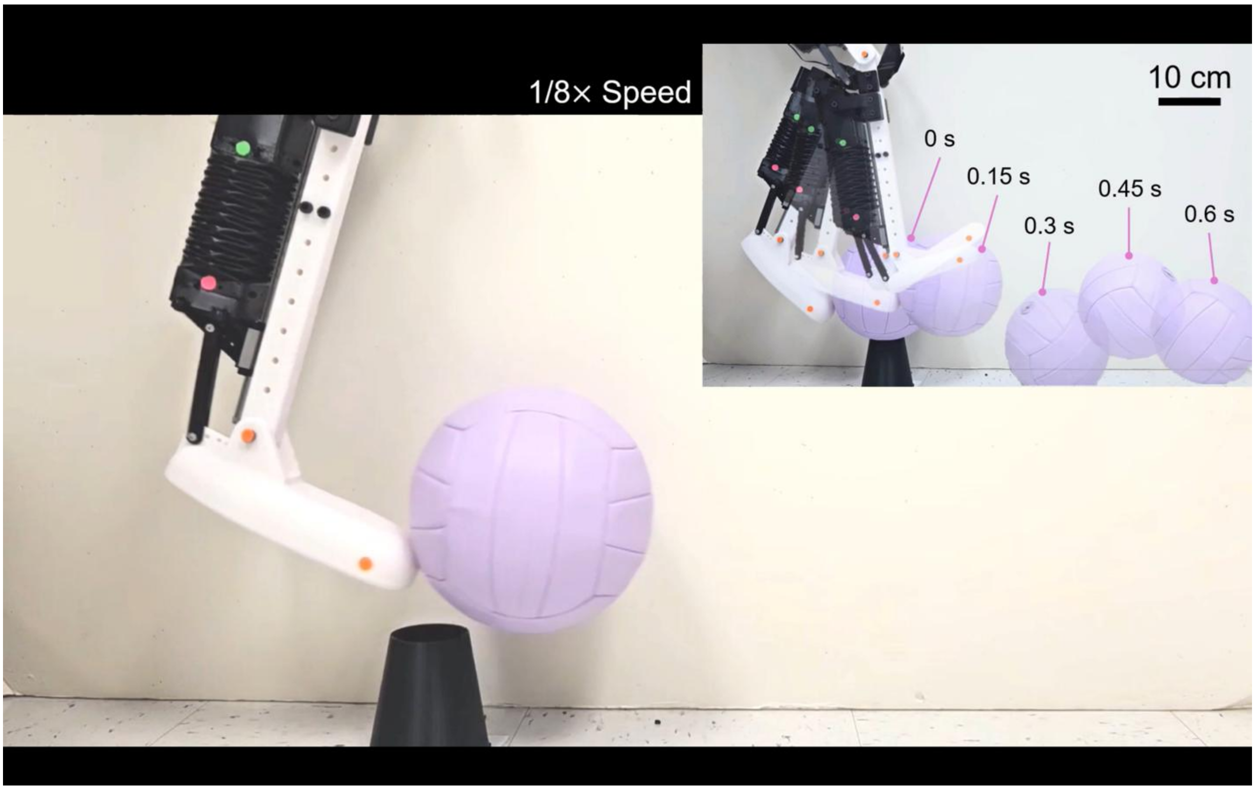

Movie S15 (.mp4 format). Artificial Musculoskeletal System – Ball Kicking Demonstration (real-time and 1/8× playbacks). The leg kicks a ball off a stand as the knee and ankle were actuated to full extension and dorsiflexion, respectively. The servos were operated at maximum operation speed.

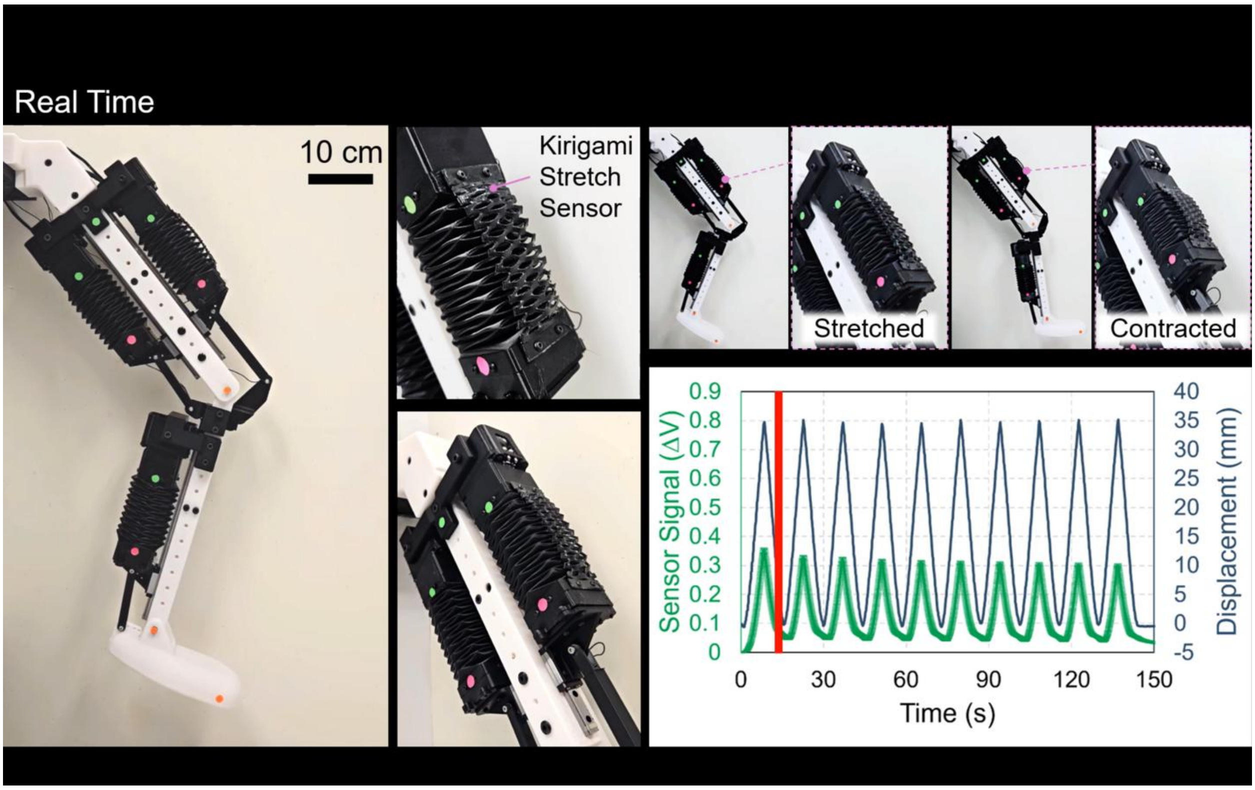

Movie S16 (.mp4 format). Artificial Musculoskeletal System – Proprioceptive Sensing (real-time playback). The Kirigami stretch sensor's voltage signal is provided over time as the quad actuator repeatedly extends to a 35-mm displacement for ten cycles.

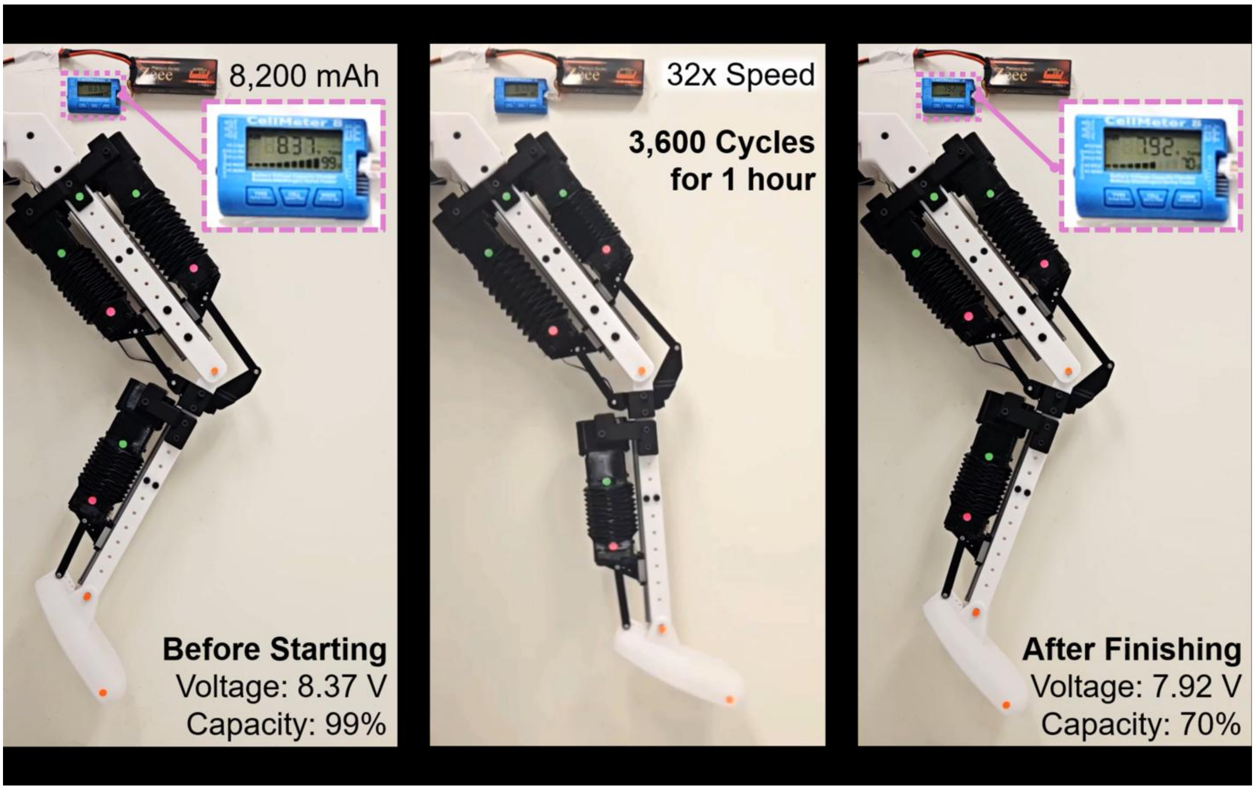

Movie S17 (.mp4 format). Artificial Musculoskeletal System – Cyclic Knee Motion with a Battery (32× playback). Leg motion was powered by a 8,200 mAh LiPo battery (99% charged, initial voltage of 8.37V). The leg bent its knee at ~1 Hz for 1 h, bending for thousands of cycles as the quad and hamstring actuators operated at full servo rotation.
